# Dynamics of COVID-19 under social distancing measures are driven by transmission network structure

**DOI:** 10.1371/journal.pcbi.1008684

**Published:** 2021-02-03

**Authors:** Anjalika Nande, Ben Adlam, Justin Sheen, Michael Z. Levy, Alison L. Hill

**Affiliations:** 1 Program for Evolutionary Dynamics, Harvard University, Cambridge, Massachusetts, United States of America; 2 Department of Biostatistics, Epidemiology and Informatics, University of Pennsylvania, Philadelphia, Pennsylvania, United States of America; 3 Institute for Computational Medicine, Johns Hopkins University, Baltimore, Maryland, United States of America; University of Notre Dame, UNITED STATES

## Abstract

In the absence of pharmaceutical interventions, social distancing is being used worldwide to curb the spread of COVID-19. The impact of these measures has been inconsistent, with some regions rapidly nearing disease elimination and others seeing delayed peaks or nearly flat epidemic curves. Here we build a stochastic epidemic model to examine the effects of COVID-19 clinical progression and transmission network structure on the outcomes of social distancing interventions. Our simulations show that long delays between the adoption of control measures and observed declines in cases, hospitalizations, and deaths occur in many scenarios. We find that the strength of within-household transmission is a critical determinant of success, governing the timing and size of the epidemic peak, the rate of decline, individual risks of infection, and the success of partial relaxation measures. The structure of residual external connections, driven by workforce participation and essential businesses, interacts to determine outcomes. We suggest limited conditions under which the formation of household “bubbles” can be safe. These findings can improve future predictions of the timescale and efficacy of interventions needed to control second waves of COVID-19 as well as other similar outbreaks, and highlight the need for better quantification and control of household transmission.

## Author summary

Social distancing is the main tool used to control COVID-19, and involves reducing contacts that could potentially transmit infection with strategies like school closures, work-from-home policies, mask-wearing, or lockdowns. These measures have been applied around the world, but in situations where they have suppressed infections, the effect has not been immediate or consistent. In this study we use a mathematical model to simulate the spread and control of COVID-19, tracking the different settings of person-to-person contact (e.g. household, school, workplace) and the different clinical stages an infected individual may pass through before recovery or death. We find that there are often long delays between when strong social distancing policies are adopted and when cases, hospitalizations, and deaths peak and begin to decline. Moreover, we find that the amount of transmission that happens within versus outside the household is critical to determining when social distancing can be effective and the delay until the epidemic peak. We show how the interaction between unmitigated households spread and residual external connections due to essential activities impacts individual risk and population infection levels. These results can be used to better predict the impact of future interventions to control COVID-19 or similar outbreaks.

## Introduction

In less than a year the novel coronavirus SARS-CoV-2, the causative agent of COVID-19, has spread from an initial foci in Wuhan, China to nearly every corner of the globe. At the time of writing, over 2 million deaths had been reported, which will likely make this emerging virus the top infectious cause of death in 2020. Several clinical and epidemiological features of COVID-19 have contributed to its disastrous effects worldwide. The overlap in symptoms with many endemic and milder respiratory infections—such as influenza, parainfluenza, respiratory syncytial virus, and seasonal coronaviruses—make syndromic identification of cases difficult. The relatively high percentage of infected individuals who require hospitalization or critical care compared to seasonal respiratory infections has put an unprecedented burden on the healthcare systems of hard-hit regions. The important role of presymptomatic and asymptomatic individuals in transmitting infection makes symptom-based isolation less effective. Uncertainty about the case fatality risk from COVID-19 [1] and misguided comparisons to seasonal influenza contributed to sluggish responses in many regions, in contrast to previous outbreaks of SARS and MERS.

In the absence of either a vaccine or antiviral therapy, and given the continuing limitations in testing capacity in most regions, the main tools implemented worldwide to control the spread of COVID-19 have been “non-pharmaceutical interventions” including “social distancing”, isolation of cases, and quarantine of contacts. All of these measures are crude attempts to prevent the person-to-person contact that drives the transmission of respiratory infections, and have been used since antiquity in attempts to control outbreaks of plague, smallpox, influenza, and other infectious diseases [2,3]. Social distancing is a blanket term covering any measure that attempts to reduce contacts between individuals, without regards to their infection status. Within two weeks of identifying the original outbreak in Wuhan, a *cordon sanitaire* had been implemented around the entire Hubei province, prohibiting travel in or out of the region and requiring individuals to remain in their houses except to buy essential supplies. Elsewhere schools and universities have been closed, international travel has been limited, restaurants and retailers shuttered, mask-wearing encouraged or required, and stay-at-home orders put in place.

Mathematical models of COVID-19 transmission provided early support for the idea that social distancing measures could “flatten the curve” and reduce the potential for COVID-19 cases to overwhelm healthcare resources. An influential report from the Imperial College COVID-19 Modeling Team showed that suppression of the epidemic to levels low enough to avoid overflow of healthcare capacity would require an “intensive intervention package” that combined school closures, case isolation, and social distancing of the entire population, applied for the majority of time over two years [4]. Kissler *et al* also came to the conclusion that large sustained reductions in the basic reproductive ratio R_0_ (the average number of secondary infections generated by an infected individual) would be needed, even after accounting for the potential role of seasonality in transmission [5]. Many more forecasting models predicted dramatic decreases in the burden of COVID-19 if interventions were enacted (e.g. [6,7]). Real-time and retrospective analyses of the growth rate of cases and deaths have suggested that in some settings the epidemic eventually slowed after the implementation of strong social distancing measures (e.g. in Wuhan and other Chinese cities [8,9], in Hong Kong [10], across European countries [11], French regions [12], or some US states [13,14]).

**Fig 1:**
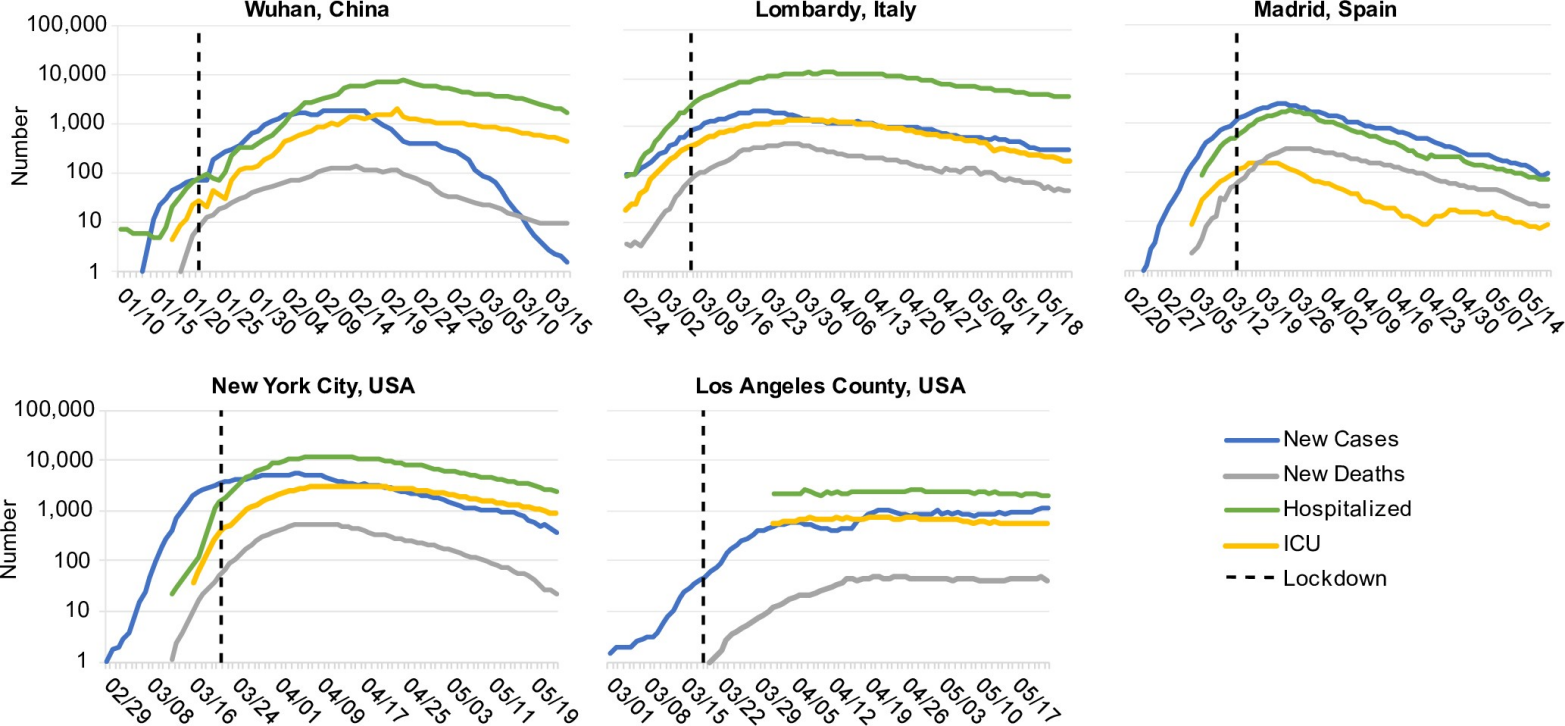
COVID-19 dynamics before and after lockdown interventions in five example regions. A) The city of Wuhan, China (8.5K km^2^, 11.1M ppl), B) The Lombardy region of Italy (23.8K km^2^, 10.1M ppl) C) The autonomous Community of Madrid in Spain (8.0K km^2^, 6.6M ppl) D) New York City in the state of New York, USA (1.2K km^2^, 8.2M ppl). E) The county of Los Angeles, California, USA (4.7K km^2^, 9.8M ppl). “New cases” and “New deaths” are daily numbers of new reports, averaged over a 7 day window centered on the current day. For Lombardy, New York, and Los Angeles, “Hospitalized” and “ICU” are the total number of patients currently in regular hospital care or critical care, respectively. In Wuhan, the same time series are the number of patients currently categorized as having “severe” or “critical” infection (using the same definitions as in our model). In Madrid, due to data availability, these series are instead the daily number of new admissions (with 7-day smoothing).

The observed dynamics of COVID-19 outbreaks following social distancing policies have been inconsistent, unpredictable, and the source of much confusion and debate in the general public and among epidemiologists. Declines in cases and deaths have not occurred uniformly across regions and have often only occurred after a long delay (**Fig 1**). The economic and social costs of these measures are immense: unemployment has surged, stock markets have plummeted, delivery of healthcare for non-COVID-19 conditions has been interrupted [15–19]. Social isolation also brings on or exacerbates mental health conditions. Weeks after implementing strong interventions, many regions have continued to see increases in daily diagnoses and deaths. Does this mean the interventions are not working? Since the political will to sustain strict social distancing measures is waning in many places, it is important to understand the expected timescale to judge success or failure. If stronger interventions—such as “shelter-in-place” orders or institutional isolation of mild cases—are needed to slow spread, when will we know this? What epidemiological and demographic features impact the timescale for epidemic waning, and how can we better predict the required duration of these measures for future outbreaks?

Social distancing measures reduce potentially-transmissive contacts occurring in schools, workplaces, social settings, or casual encounters, but they generally do so by confining individuals to their households without additional precautions. Thus, we would expect that the impact of social distancing measures might depend on the relative contribution of within-household transmission to disease spread, the distribution of household sizes, the number of households containing at least one infected individual at the time an isolation measure is enacted, and the amount of residual contact between households for the duration of the intervention. What do we know about these factors for COVID-19 or respiratory infections more generally, and how do they interact to determine epidemic dynamics after an intervention?

In this paper we examine the impact of COVID-19 clinical features and transmission network structure on the timing of the epidemic peak and subsequent dynamics under social distancing interventions. Using data from large-scale cohort studies, we parameterize a model tracking the progression of COVID-19 infection through different clinical stages. We combine this with data-driven transmission networks that explicitly consider household vs external contacts and how they are differentially altered by social distancing measures. We consider various scenarios for the efficacy of interventions in reducing contacts, heterogeneities in their adoption in different demographic groups, the relative role of transmission in different settings, and the timing of partial or complete relaxation of isolation measures. We evaluate both population-level outcomes as well as determinants of individual risk of infection. Our results show that even following the implementation of strong social distancing measures, the epidemic peak can occur weeks to months later, and the decline in cases can be extremely slow. The efficacy of within-household transmission plays a critical role in the timescale and overall impact of these measures. These findings provide an impetus for continued adherence to social distancing measures in the absence of immediate results, can inform planning for hospital capacity, and suggest that retrospective efforts to assess the efficacy of different intervention policies should account for these expected delays.

## Methods

### Modeling the spread and clinical progression of COVID-19

**Fig 2:**
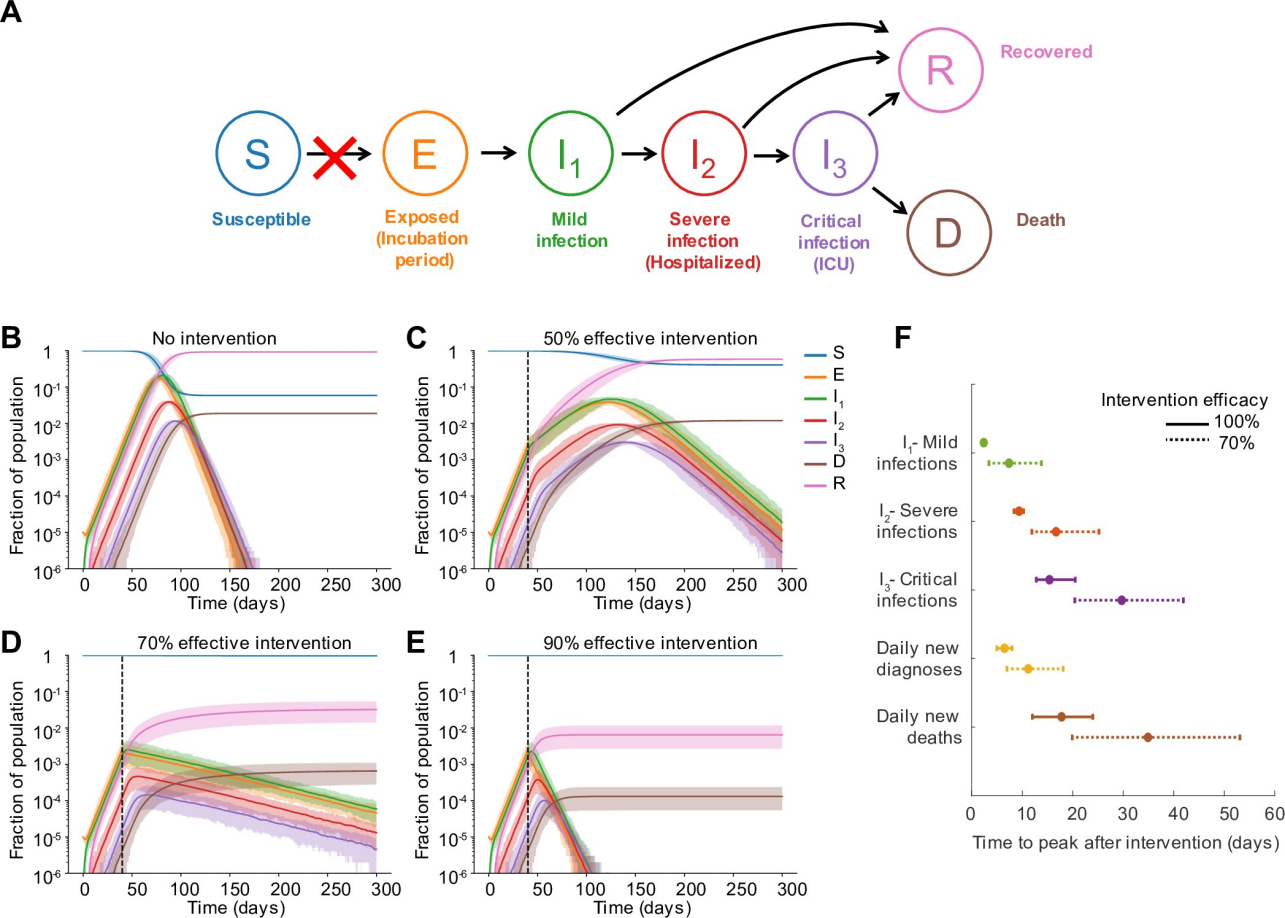
Dynamics pre and post social distancing intervention in well-mixed populations. A) Model of COVID-19 clinical progression and transmission. The model is described in the text and detailed in the Methods. Social distancing interventions (red X) reduce the rate of transmission and the generation of new infections. B-E) Simulated time course of the population level prevalence of each clinical stage of infection under different intervention efficacies. The intervention was implemented on day 40. Solid line is mean and shaded areas are 5th and 95th percentile. Black dotted line shows the time the intervention began. F) Time to peak of different infection stages, measured as days post-intervention. The first three quantities are peak prevalence levels (I_1_, I_2_, I_3_), while the latter two are peak daily incidence values. We assume that cases are diagnosed only at the time of hospitalization. Daily incidence values were first smoothed using moving averages over a 7 day window centered on the date of interest. Bars represent 5th and 95th percentile.

We modified the classic SEIR compartmental epidemiological model to describe the dynamics of COVID-19 infection (**S1 Text** and **Fig 2A**). After infection, individuals pass through an ~ 5 day incubation period before developing asymptomatic or mild infection, which could include fever and cough or other symptoms. This stage lasts ~ 1 week and individuals are infectious for this duration. A portion of individuals progress to “severe infection”, which is typically characterized by pneumonia requiring hospitalization, and we assume averages 6 days. Some individuals progress further to “critical infection”, which requires ICU-level care that often includes mechanical ventilation, and some of these individuals eventually die (after ~ 8 days of critical care), leading to an ~2% case fatality risk. At each stage, individuals who don’t progress or die, recover and are assumed to be immune for the duration of the outbreak. The duration of each stage of infection is assumed to be gamma-distributed with mean and variance taken from the literature. Infectious individuals can transmit to any susceptible individuals with whom they are in contact, with a constant rate per time for the duration of their infection. With our baseline parameters, the doubling time of infection is ~ 4 days, the basic reproductive ratio R_0_ is ~3, and the serial interval is ~8 days, in agreement with epidemiological studies of COVID-19. A detailed description of the clinical definitions of different infection stages, the model behavior, and the model parameters and references are given in the Methods.

**Fig 3:**
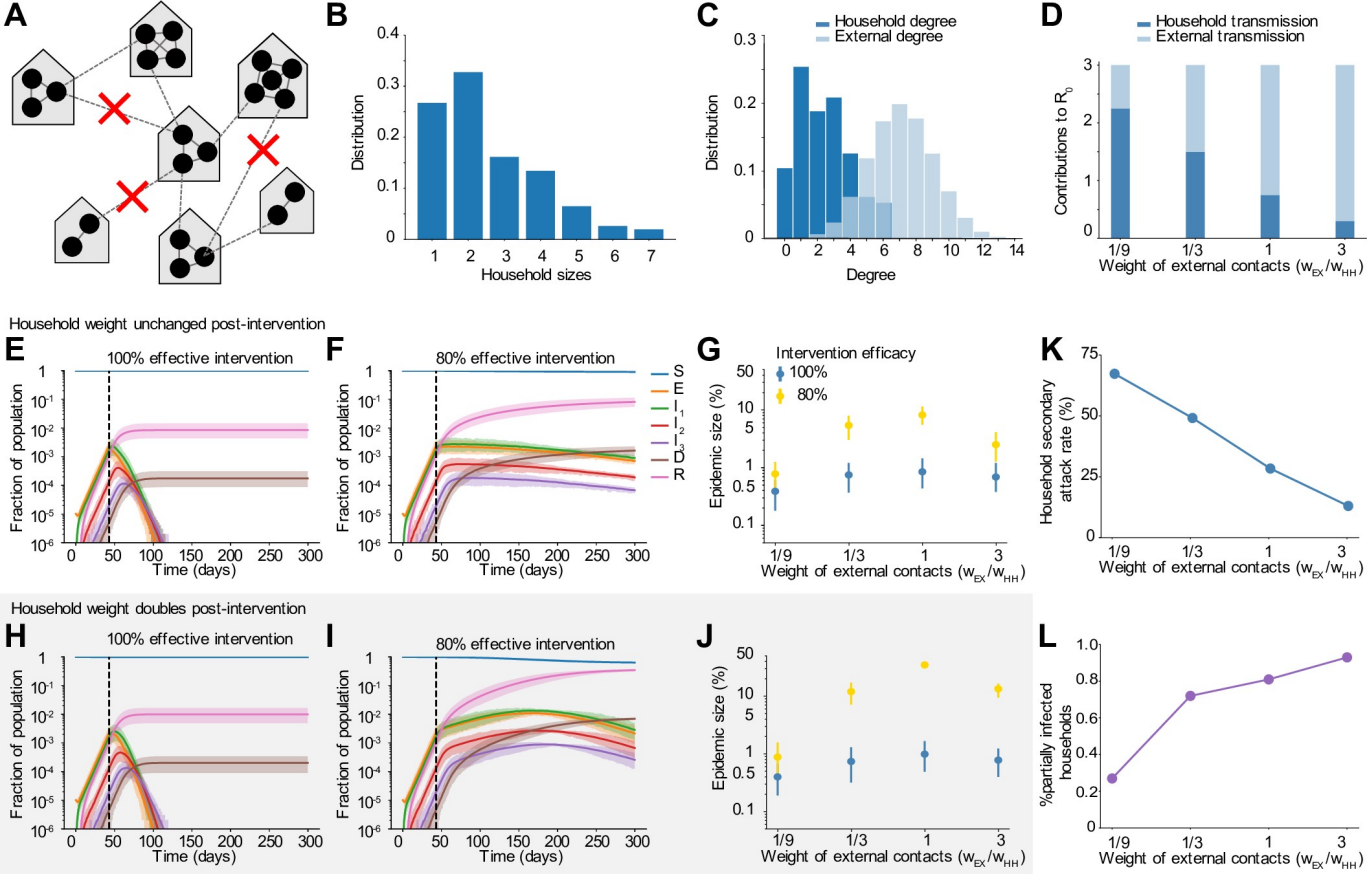
Dynamics pre and post social distancing interventions in network-structured populations with household and external transmission. A) Multi-layer network of transmission. Individuals have contacts within their households and with others outside the household. Household and external contacts may have different weights (e.g. different likelihood of transmission), due to for example different levels of physical contact or time spent together per day. Social distancing interventions (red X) remove or decrease the weight of external contacts. B) Distribution of household sizes. C) Distribution of the # of contacts (degree) within the household and outside the household. D) The contribution of household and external spread to the total R_0_ value as a function of the relative weight of external contacts. E)-F) Simulated time course of different clinical stages of infection under an intervention with efficacy of 100% (E) or 80% (F) at reducing external contacts, when household and external contacts have equal weight. Black dotted line shows the time the intervention began. G) The role of the relative importance of household vs external contacts in determining the outcome of the intervention, measured by the size of the epidemic. Epidemic final size is defined as the percent of the population who have recovered by day 300. H-J) Same as above but under the scenario where the weight of household contacts doubles post-intervention (*w*_*HH*_ → 2*w*_*HH*_, due to increased time spent in house). K) The household secondary attack rate, defined as the probability of transmission per susceptible household member when there is a single infected individual in the house, as a function of the relative weight of external contacts. L) The percent of households which are “seeded” with infection at the time the intervention was implemented (i.e. have at least one infected individual). In all scenarios the overall infection prevalence at the time intervention was started was identical.

We then simulate infection spreading stochastically through a fixed, weighted contact network with one million nodes. The population size is chosen to represent a typical metropolitan area. As a baseline scenario, we consider a simple approximately well-mixed population where anyone can potentially transmit the virus to anyone else in the population. To more accurately capture human contact patterns, and how they are altered by social distancing measures, we constructed multi-layer networks describing connections within households and external connections (**S1 Text** and **Fig 3A**). Each individual was assigned to a household and connected to everyone in their house. Household size distributions were taken from the 2010 United States census (average household size *n*_*HH*_~2.5, full distribution shown in **Fig 3B**). External connections were constructed by connecting individuals to people in other households. The distribution of the number of external connections was taken from detailed contact surveys that recorded daily interactions amenable to transmission of respiratory infections (average *n*_*EX*_~7.5, standard deviation 2.5) [20,21]. As a baseline case we constructed “two-layer” networks assuming these external connections were random, whereas later in the paper we consider more complex and realistic “five-layer” network structures. While these data sources inform the *number* of contacts, the probability of infection depends both on the number of unique contacts and on the time spent together and the intensity of the contact, which can be represented by weights in the network. We hypothesized that household and external contacts could have different effective weights. For example, individuals may spend 8–10 hours a day with coworkers or classmates, but only a few waking hours with household members, and so external contact could have higher weights. Alternatively, individuals may have more intense physical contact with household members, such as children or spouses with whom co-sleeping can occur. Since these weights are unknown, we considered a range of scenarios for the relative weights of household (*w*_*HH*_) and external (*w*_*EX*_) contacts, keeping the total transmission intensity (basic reproductive ratio *R*_*0*_) constant. These scenarios result in different observed values of the household “secondary attack rate” (the probability a single index individual infects any given household contact) (**Fig 3K**). We also hypothesized that when individuals are isolated in their homes as a result of social distancing measures (e.g. school closures or work-from-home mandates), they may be spending significantly more time with household members and thus have a higher transmission rate. We modeled this by allowing the weight of household contacts to increase during an intervention.

We model the implementation of social distancing measures by reducing the weight of all external contacts (or all contacts in the well-mixed model) by a fixed % that we term the “intervention efficacy”. Alternatively, we could randomly remove a fixed % of contacts, but the results are very similar (see **Methods**). Our model is similar to other models that have been used to describe the spread of COVID-19. A unique feature of our model is that it simultaneously captures the clinical progression of COVID-19 (as opposed to simpler SEIR models), a reasonable approximation of contact network structure (as opposed to well-mixed models), and realistic distributions of the durations of states (as opposed to continuous-transition models which assume exponentially-distributed durations, and lead to unrealistically long tails in infection after strong interventions). We can simulate infections for the duration of the epidemic in less than 1 minute on a single GPU, in populations of a million.

## Results

### Observed COVID-19 dynamics following social distancing interventions

To characterize the dynamics of COVID-19 following social distancing measures, we chose five regions from around the world with large outbreaks: the city of Wuhan, China, the Lombardy region of Italy, the Community of Madrid in Spain, New York City in the state of New York, USA, and the county of Los Angeles, California, USA (**Fig 1**). These regions each implemented strong “lockdown” measures (aka “stay-at-home” or “shelter-in-place” orders) within 3 weeks of their first reported COVID-19 case and provided data not just on cases and deaths but also on cases requiring hospitalization and ICU-level care (see **S1 Text**). In each setting, there was a long delay between the implementation of social distancing and the peak incidence of cases (1.5–3 weeks) and deaths (2–3 weeks), or peak occupancy in hospitals and ICUs (~1 month). The timescale of the eventual decline in cases post-peak was much slower than the initial increase in cases in all regions, with a half-life between 10 and 24 days in all regions except Los Angeles, where the outbreak approximately plateaued but did not begin decreasing. The goal of this paper was to understand whether the clinical progression of COVID-19 and transmission network structure could explain these types of post-intervention dynamics.

### Prolonged clinical progression of COVID-19 leads to delay until decline in cases and deaths following an intervention

We first considered the role of the clinical features of COVID-19 alone, in the delay from implementation to peak infections and deaths, by simulating our model in an unstructured population. The intervention was implemented when cumulative reported cases were ~200 per million and deaths ~5 per million (total infected ~1%), mirroring the timing of stay-at-home orders across major US metropolitan areas (see **S1 Text**). While we expect the number of new infections to begin decreasing immediately, newly infected individuals in the “exposed class (E)” (incubation period) cannot generally be tracked, since they are pre-symptomatic and often not yet shedding enough virus to test positive. Instead, later stages of infection are monitored.

We found that under a perfect intervention, we expect ~2 days delay until the peak prevalence of mild infections, ~9 days for severe infections, and ~15 days for critical infections, suggesting that the requirements for healthcare capacity may peak quite a bit after implementation (**Fig 2**). In the more realistic scenario where the intervention is imperfect (70% effective), these timelines are significantly extended, for example to ~7, 17, and 30 days for mild, severe, and critical infections respectively. In most regions, individuals are reported at the time of diagnosis, and not tracked until recovery, and so case counts can only be used to track *incidence* rates, not prevalence levels. We consider a region where infections are only counted upon hospitalization (progression to severe class), and then find that peak incidence of cases occurs 7 and 11 days after an intervention that is 100% or 70% effective. Daily deaths peak much later: after 18 days (100% effective) to 35 days (70% effective). Under our parameter values, a 50% intervention “flattens the curve” but does not prevent spread, and incidence cases and deaths don’t peak until 13 and 15 weeks after the intervention, respectively. The total percent of the population infected over the course of the whole epidemic was reduced from ~92% to ~0.6% with a 100% effective intervention, but only to 58%, 3%, or 0.65% with a 50, 70% or 90% effective intervention.

The exact timings that we report here depend on the assumptions of our model, in particular, the average duration of each stage of infection (see **S1 Text** for details) as well as on the epidemic growth rate pre-intervention (it takes longer for epidemics that were growing faster to peak and begin declining). However, the qualitative finding that peaks in case counts, hospitalizations, and deaths can be significantly delayed beyond when an intervention is implemented is a general finding for models tracking the natural history of COVID-19. Note that in our model, we assume that the intervention is adopted the same day it is instituted, whereas in reality, there may be a further delay until individuals are able to comply with the intervention.

### The relative contribution of household and external spread influences outcome of interventions

We hypothesized that the continual spread of COVID-19 within households after the implementation of social distancing measures could further delay peak cases and deaths, and increase the number of people infected despite the intervention. Using our network-structured model (see **Methods**) for household and external contacts, we simulated the implementation of interventions of increasing efficacy under different assumptions about the relative weight of the household vs external contacts. In addition, we examined the impact that the increased time spent with household members (and hence an increased transmission potential) after stay-at-home policies begin could have on the outcome of an intervention and the timescale for disease elimination (**Fig 3**).

With our baseline assumption that household and external contacts had equal weight, we observed that cases declined rapidly under very strong interventions (**Fig 3E and 3H**), while imperfect interventions (e.g. ~80%) often resulted in very gradual decreases in cases over many months (**Fig 3F**). In both scenarios the eventual fraction of the population infected was dramatically reduced compared to the no intervention case, but these long timescales likely mean that costly social distancing policies cannot be maintained long enough for suppression of the epidemic to occur. This slow decline could be further compromised if the risk of transmission within a household increases under stay-at-home policies (**Fig 3I**). In this case the epidemic could continue to increase for months post-intervention before eventually declining, albeit still to a much lower final size than in the absence of interventions.

When the outcome of an intervention was measured by the total fraction of the population infected over the course of the outbreak, we found that there was a surprisingly complex relationship between the relative contribution of household and external contacts to transmission, and the intervention success (**Fig 3G and 3J**). Keeping the total R_0_ constant, social distancing interventions are most effective when either external contacts have very high weights or when they have very low weights. In the former case (high external weight + low household weight), most of the pre-intervention transmission comes from outside the household, and the intervention is very effective at blocking this transmission (**Fig 3D and 3K**). At the time the intervention is implemented, many households are “seeded” with infections that originated outside the house (**Fig 3L**), but after the intervention, household transmission alone is not effective enough to lead to a new generation of infections in most houses, without seeding from the outside (i.e intervention efficacy <100%). When external contacts have low weight, the intervention is highly effective but for a different reason. Most transmission is inside the household and can continue post-intervention (**Fig 3D and 3K**), but very few households are seeded with infections (**Fig 3L**). The weak inter-household contacts are further weakened by the intervention and spillover between households is unlikely, meaning that the infection quickly burns through susceptibles within a household then dies out.

In the intermediate regime, where household and external contacts have approximately equal weight, social distancing interventions are less effective, and are very sensitive to imperfect efficacy. For example, when external contacts have ~1/3 the weight of household ones, each type of contact contributes equally to the overall pre-intervention *R*_*0*_ (since there are ~3x the number of external contacts as household ones). With a 100% effective intervention, the final epidemic size is ~0.7%, but rises to ~7% with a 80% effective intervention (**Fig 3G**). The combination of enough household spread (*R*_*0*_^*HH*^ *>1*) to allow efficient transmission post-intervention within “seeded” households and enough external spread (*R*_*0*_^*EX*^ *>1*) to seed households before the intervention is implemented to allow post-intervention spillover of infections to other households is the most difficult case for control. These effects are exacerbated if we assume household transmission rates (contact weights) can increase post-intervention (**Fig 3J**). For an 80% effective intervention, the final epidemic size can be 5–10—fold higher than expected due to increased chance of within-household transmission. We repeated these simulations with a hierarchically-clustered external layer (see **Methods**) to check the robustness of the trends to details in the large-scale clustering of the transmission network (**S1 and S2 Figs**). We found the trends to be preserved, with the most noticeable difference being in the number of houses “seeded” with infection at the time of intervention.

### Residual household transmission can further delay time to see the impact of an intervention

**Fig 4:**
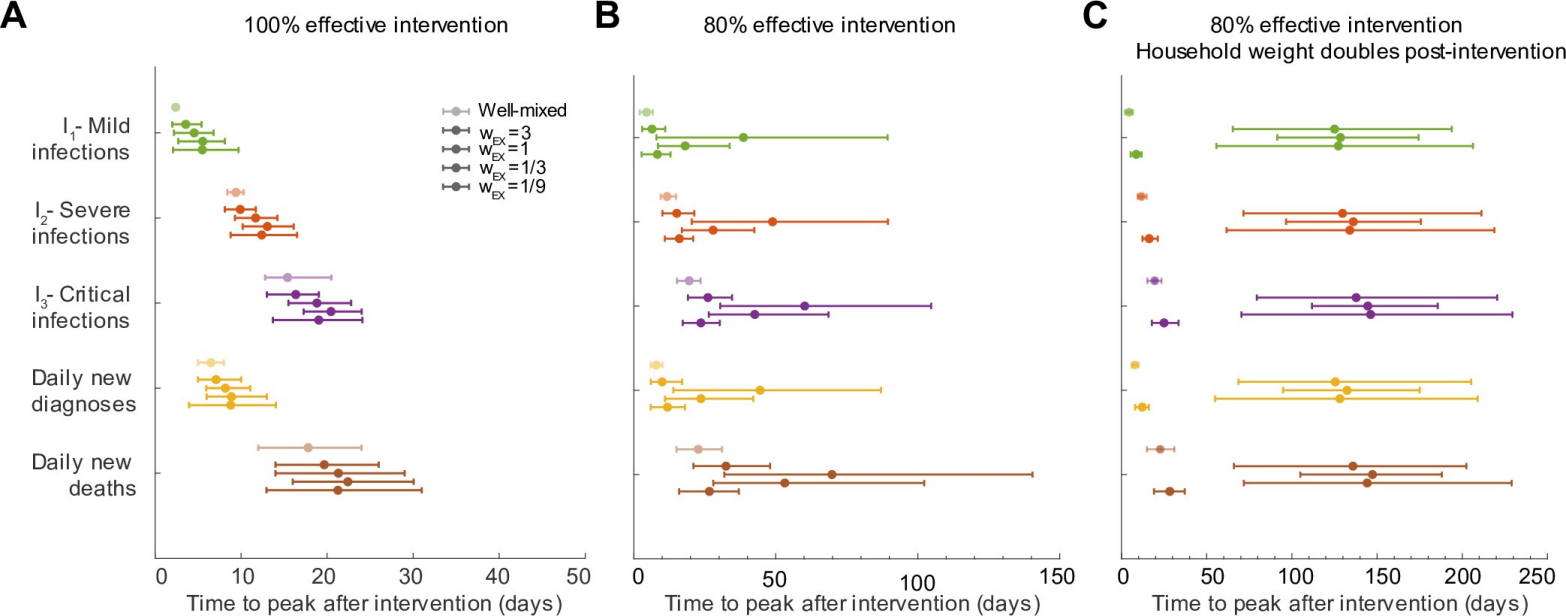
Time to epidemic peak after social distancing interventions depends on the relative roles of household and external transmission. A-C) Time to peak of different infection stages, measured as days post-intervention. A) Social distancing intervention with 100% efficacy at reducing external contacts (or all contacts in the case of a well-mixed network). B) Social distancing intervention with 80% efficacy. C) Social distancing intervention with 80% efficacy, and assuming that household weights double post-intervention (*w*_*HH*_ → 2*w*_*HH*_, due to increased time spent in the home). The first three quantities are peak prevalence levels (I_1_, I_2_, I_3_), while the latter two are daily incidence values. We assume that cases are diagnosed only at the time of hospitalization. Daily incidence values were first smoothed using moving averages over a 7 day window centered on the date of interest. Bars represent 5th and 95th percentile. For each clinical stage included (each different color), the lighter-colored data point is the comparison to the well-mixed population, then the other points are for decreasing contributions of external connections and increasing role of household transmission.

We found that the expected time to peak infections and deaths after a social distancing intervention was implemented could be increased dramatically when we accounted for household structure, and was sensitive to the relative importance of household and external contacts before and after the intervention (**Fig 4**). Under a 100% effective intervention (**Fig 4A**), the delays to peaks were driven mainly by the clinical progression alone, similar to the case of the well-mixed population, but were slightly extended due to residual spread restricted to a single household. In simulations it took around 2 weeks until peak hospitalizations and 3 weeks to peak critical care cases or daily deaths. However, under an imperfect but still strong intervention (e.g. 80% effective), the times to peak were much longer and sensitive to the relative weights of the external and household contacts (**Fig 4B**). Delay to peak cases was longest in the intermediate regime where external and household contribution to transmission was approximately equal. For example, when external and household weights were equal, it took an average of ~ 5.5 weeks to reach peak cases with mild symptoms, ~ 7 weeks until peak cases hospitalized with severe infection, and ~ 8.5 weeks to the peak of cases in critical care. The daily incidence of new deaths didn’t peak for ~ 10 weeks.

The delays in time to peak were less extreme if external contacts had very high or very low weights relative to the weight of household contacts (**Fig 4B**). In the case of very high external weight, most individuals were infected from contacts outside their household before the intervention (**Fig 3L**). Household spread is relatively inefficient, and has only a minor contribution to the baseline *R*_*0*_ value (**Fig 3D**). In most households, there is no further spread after the intervention is implemented. As a result, the epidemic peaked sooner: peak daily deaths occurred an average of ~ 3 weeks post 100% effective intervention and ~ 5 weeks post 80% effective intervention. On the other hand, when external weight is a lot lower as compared to the household, only a small fraction of households are seeded with infection by the time intervention is started (**Fig 3L**). Intervention is very effective at suppressing external transmission and so, even though household transmission continues during intervention it can not spill over between households. This causes the epidemic to peak sooner as susceptibles in households get infected quickly and then the infection dies out. On average, peak daily deaths occurred ~ 3 weeks (100% effective intervention) and ~ 4 weeks (80% effective intervention) post intervention.

These results were exacerbated if we assumed that the importance of household contacts increased post-intervention (**Fig 4C**), due to increased time spent in close quarters. In that case, peaks increased to up to ~ 6 months for cases in critical care and daily deaths under an 80% intervention. With higher household weights, the efficacy of spread within a household was stronger, making new generations of infection post-intervention very likely to occur in households with at least one case. Then, these household infections are more likely to spill over into other households, even when most external contacts are eliminated by the intervention. Together, these effects allow for multiple generations of transmission to persist even after a strong intervention.

In many regions around the world, the effect of social distancing interventions is monitored in real-time using estimates of the time-dependent reproduction number *R*_*t*_ (e.g. [22]). We applied standard procedures for calculating *R*_*t*_ [23] to the incidence data from our simulations, and using the time at which *R*_*t*_ first crossed the threshold of 1 as a measure of the delay, we found that the trends agreed with those reported for the epidemic peak (**S8 Fig**).

### Clustered adoption of social distancing measures can further compromise efficacy

Our results so far have assumed that external contacts in the transmission network are random connections between pairs of individuals in the population, and that a social distancing intervention results in a uniform random reduction or deletion of these connections. In reality, human contact networks tend to be highly structured, with groups of individuals with high levels of interconnectedness and large variation between individuals in total contacts (e.g. [24,25]). Moreover, we don’t necessarily expect adherence to social distancing measures to be random. For certain occupations or in certain demographic groups, individuals are less likely to be able to work-from-home or otherwise reduce contacts outside the home. This can lead to clusters of individuals among whom contacts remain high despite interventions. We hypothesized that this clustered adoption of social distancing measures could lead to more residual transmission, longer times to peak cases and deaths, and longer times to eradicate infection from a given region.

**Fig 5:**
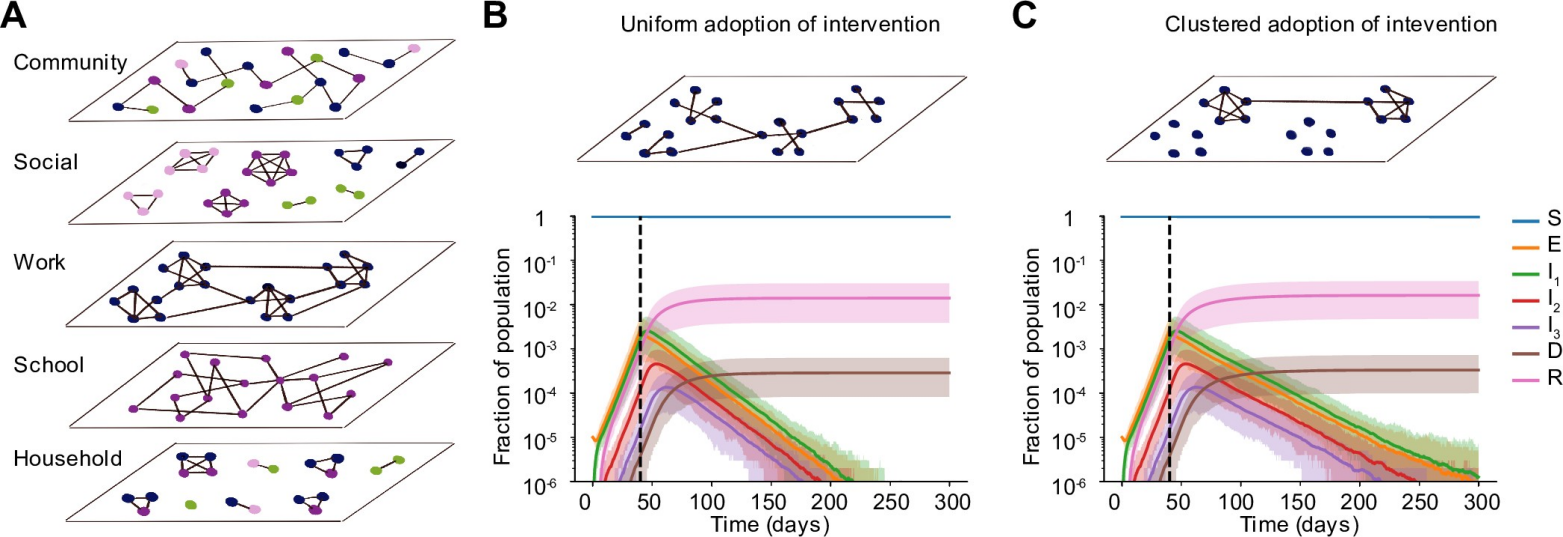
Clustered vs uniform adoption of social distancing measures. A) Schematic of the multi-layer network created to more realistically capture non-household contacts and how they are altered by social distancing measures. In each layer, the degree distribution and level of clustering were chosen to match data. The “community” layer represents any other contact not fitting in the other four categories. Colors of nodes represent four broad age groups that determine network membership and structure: preschool-aged (pink), school-aged (purple), working-aged (blue) and elderly (green). B)—C) Simulated time courses of infection in the presence of social distancing intervention with random (B) vs clustered (C) adherence to measures. In both cases, all school connections were deleted post-intervention and 85% of connections were uniformly deleted at random in the social and community layers. In B) 85% of work connections were uniformly deleted whereas, in C) 85% of workplaces were dissolved, leading to clusters of disconnected vs connected individuals in the work layer of the network. The effective intervention efficacy for all layers combined was ~ 88% in both scenarios. Black dotted line shows the time the intervention began.

To examine these effects, we constructed more realistically-structured, age-segregated external contact networks. The population was divided into four broad age groups: preschool-aged, school-aged, working-aged and elderly. Based on large-scale contact surveys and other modeling studies [20,21,25–27], we broke down external contacts into four different layers—school, work, social and community (**Fig 5A**). Age groups determined network membership. School and work layers consisted of connections between individuals only belonging to the school-aged and working-aged groups respectively. Individuals belonging to all age groups were part of the social and community layers. We used a variety of data sources to construct the networks for each layer with degree distributions (both mean and variance in # of contacts) as well as levels of clustering (aka transitivity, a measure of interconnectedness) that matched data (see Methods). We assumed that during a social distancing measure, school contacts were completely removed, and that work, social, and other contacts were reduced by an amount equal to the intervention efficacy. For work contacts, we also considered the case where edges weren’t removed at random, but instead, certain “workplaces” were completely dissolved, whereas others remained (**Fig 5C**, top). With this implementation, the levels of clustering in the external network was high both before and after an intervention. Other studies have shown that such clustered adoption of preventive behavior can lead to lower than expected efficacy of vaccines and mass drug administration [28–31].

We found that when the intervention efficacy was high, most outcomes were surprisingly not worse under this clustered adoption (**Fig 5**). Time to peak cases, hospitalizations and deaths were similar under random deletion of edges (**Fig 5B**, bottom) and under the correlated deletion scheme (**Fig 5C**, bottom). However, we found that the time until infection was eliminated from the population was much longer: increasing from ~ 180 days to ~ 220 days for population sizes of a million. For a less effective intervention, the difference in outcomes for the two deletion schemes was more prominent (**S3 Fig**). Under clustered adoption, the epidemic plateaued and took much longer to decline compared to the case of uniform adoption where decline began immediately. We again performed a sensitivity analysis to check the robustness of these findings to meso-scale clustering of contacts in the network (see **Methods**) and found the trends very similar (**S4 Fig**). These findings suggest that targeting demographic groups like essential workers, where pockets of infection might persist, with more aggressive cases-based measures and contact tracing may be necessary to reach elimination goals faster.

### Individual risk of infection depends on household size and occupation

**Fig 6:**
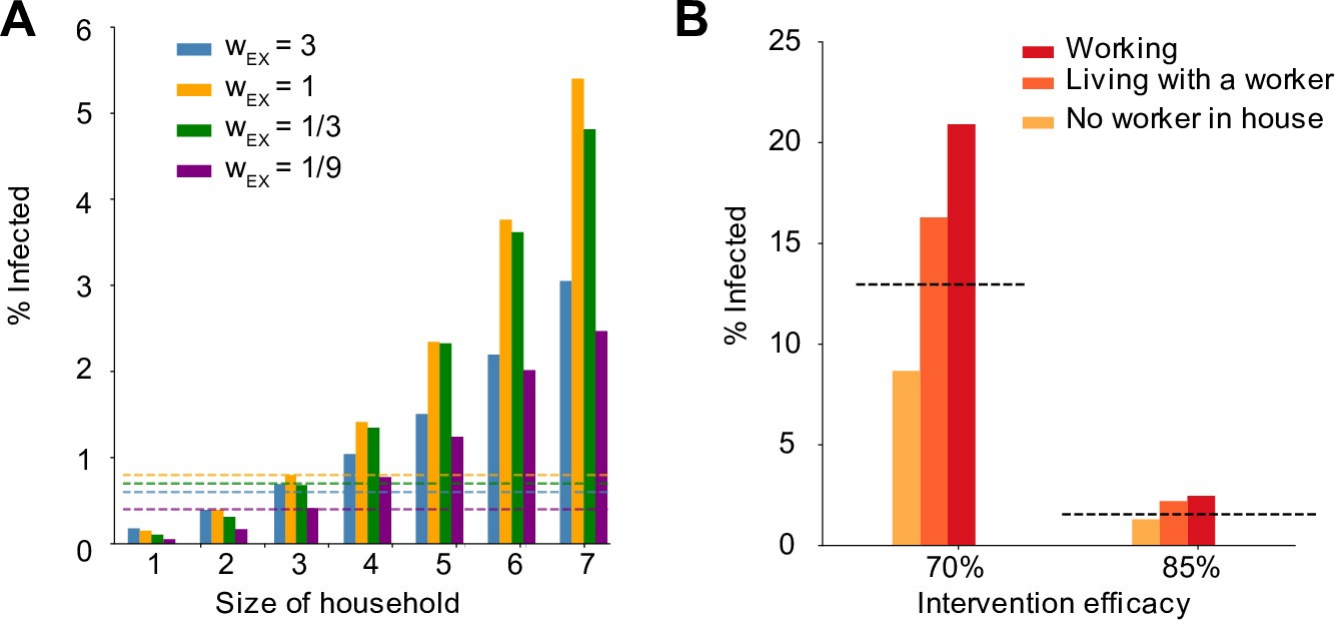
Individual risk of infection depends on household size and worker status. A) Risk of infection versus household size in simulations. Risk of infection was calculated after 300 days, with 100% intervention efficacy. Bar colors represent different relative weights of external contacts (compared to household contacts). Dotted lines are the population level average infection levels for the same scenarios. B) Risk of infection versus worker status. A “worker” is defined as someone with an occupation in which they continue to work outside the home despite social distancing measures. Categories include being a worker yourself (red), living in a household with at least one other individual who is working (orange), or having no workers in the house (yellow). As a comparison the population average risk is shown (dotted line). Interventions that reduce the overall number of people working outside the home by 70% and 85% are shown (in all cases all schools are assumed to be closed and the same percent of social and community contacts are removed).

So far our evaluations of social distancing measures have focused on population-level outcomes such as the timing of the epidemic peak and the overall fraction of the population infected. However, these findings mask significant heterogeneity in individual risk. From our simulations, we extracted the individual probability of infection as a function of household size (**Fig 6A**), as well as in relation to the external contacts maintained after an intervention (**Fig 6B**). We found that the risk of infection increased dramatically with the household size: with our baseline parameters, it ranged from <0.2% for individuals living alone to 5.4% for households of size 7 (**Fig 6A**). These differences occurred independently of the relative weight of household vs external contacts. The supra-linear increase in risk with household size is driven by the fact that in larger households there is both more risk of seeding of infection from outside, as well as more individuals to spread to within the household leading to less chance of extinction of spread.

We also examined the increased risk faced by “essential workers”, or others who maintained contacts in their “work” networks during the time social distancing measures were in place (**Fig 6B**). Under more extreme distancing (~85% reduction in contacts), the relative risk of infection among workers relative to the population average was 1.6, while for individuals not working themselves but living in the same household as someone who was working was 1.4. In comparison, individuals belonging to households with no workers had a relative risk of 0.8. For a less effective intervention (~70%), these values were 1.6, 1.3 and 0.7 respectively. These findings highlight the risk faced by communities in which larger households are common and/or in which more individuals per household may maintain external connections despite social distancing measures.

### Expanding circles can be safe partial relaxation strategies only under certain conditions

**Fig 7:**
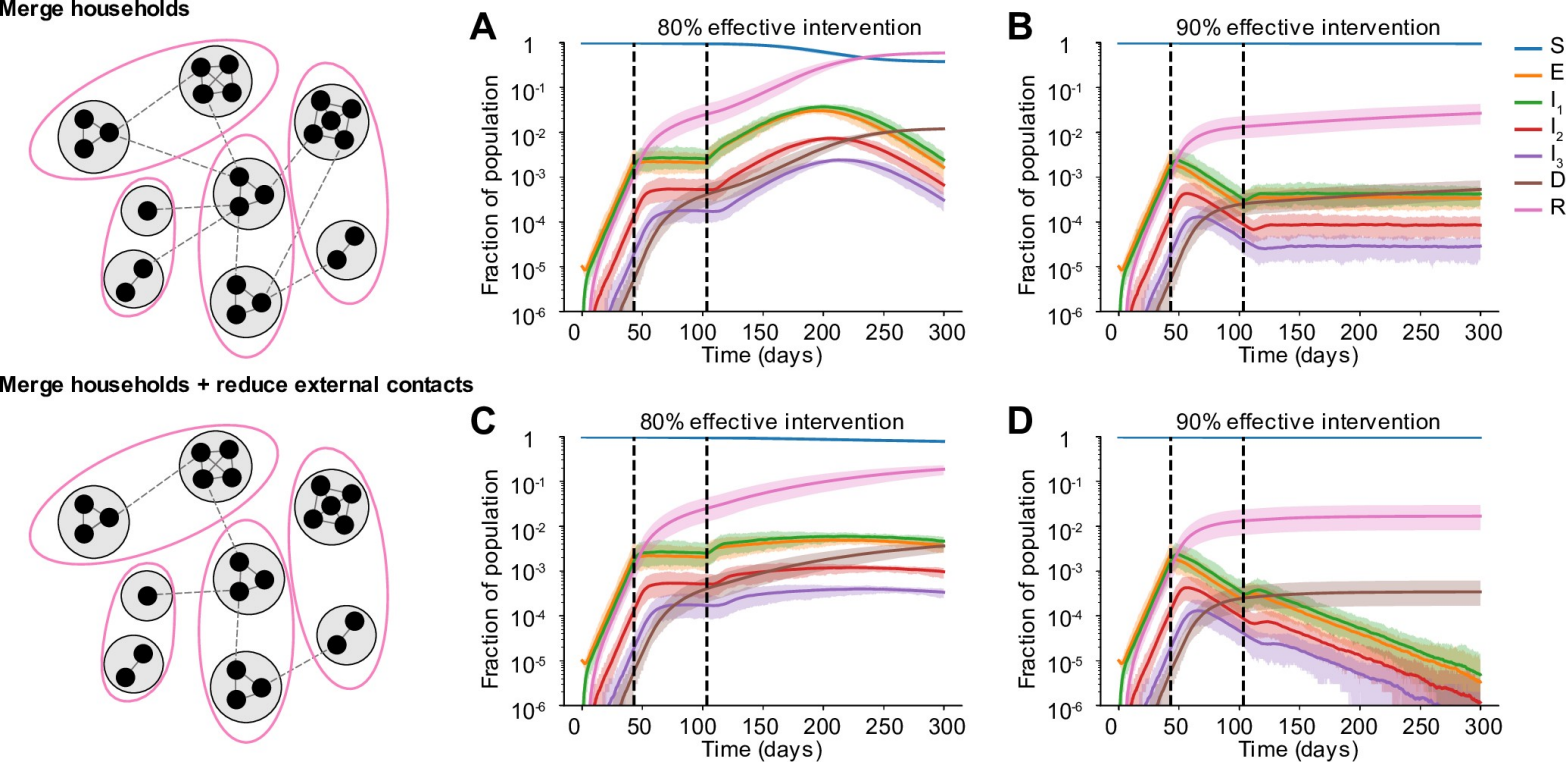
Effect of partially relaxing intervention by forming household bubbles. Some time after a social distancing intervention was implemented, each household merges with another random household. In each resulting two-household “bubble”, all individuals are connected to all other individuals. A)-D) Simulated time courses of infection before and after social distancing interventions (with 80% vs 90% intervention efficacy) and after partial-relaxation by household merging. Top row: External contacts of individuals were unchanged after two households were merged, such that overall number of contacts increased. Bottom row: External contacts for individuals were reduced after two households were merged, such that overall number of contacts remained unchanged. In all cases, intervention was started 43 days after the onset of the epidemic (first black dotted line) and was relaxed after two months (60 days, second black dotted line).

As a step towards relaxing social distancing measures in settings where the incidence of cases and deaths has stabilized or is declining, some regions are proposing partial relaxation strategies whereby groups of households merge to form larger “expanded circles” or “bubbles”, but still minimize external contacts [32,33]. Such multi-household groups could have enormous social benefits, such as providing childcare relief and improving productivity of working parents, and reducing the mental health toll of social isolation. To examine when this strategy could be safely implemented without risking a rebound in cases, we randomly joined households 1, 2, or 3 months after the implementation of a strong social distancing measure (80 or 90% effective) (**Fig 7**).

We found that these household-merging strategies could be safe only if a few criteria were met. Firstly, they must be applied in the context of steadily declining cases and deaths (**Fig 7B and 7D**). In situations where infection levels had stabilized but were barely declining, forming bubbles always led to at least some resurgence of cases which returned to or exceeded peak levels (**Fig 7A and 7C**). Secondly, household bubble formation should ideally be accompanied by a further decrease in contacts outside the house (for example, only one grocery trip per dual-family household instead of two) and a redistribution of the effective number of household contacts instead of allowing them to double (for example, by spending time with subsets of the dual household instead of all time as a complete group). Otherwise, a previously declining epidemic could instead stabilize at a persistent level (**Fig 7B**), or an otherwise stable epidemic could temporarily resurge (**Fig 7A**). When resurgence occurred it took 1–4 weeks to see noticeable increases in hospitalizations or deaths. We did not find a strong dependence on the timing of household bubble formation. As before, we also tested the robustness of these results to details in the large-scale clustering of the network by using a metapopulation model that incorporates the notion of “neighborhoods” (see Methods). We found that the trends were unaffected independent of whether the merged households belonged to the same (**S6 Fig**) or different neighborhoods (**S5 Fig**).

Clearly households with less external contacts would be at the least risk from merging with others, and these policies should only be encouraged in regions where general social distancing has clearly reduced the prevalence of infection. Similar to our findings in earlier sections, our predictions are more optimistic when household and external contacts contribute less equally to transmission.

## Discussion

Here we show that the clinical and epidemiological features of COVID-19 interact to produce long expected delays between the implementation of strong social distancing measures and when their effects become apparent. Part of the delay is clinical. After infection, individuals generally pass through an asymptomatic incubation period before entering a phase of mild or moderate symptoms, and some fraction eventually require hospitalization. Documented deaths often occur after extended stays in critical care wards. The progression from initial infection to a reportable case (often at hospital admission) or death can be weeks, and is not interrupted by current interventions. In addition, social distancing measures reduce transmission outside the household, but in general they involve isolating individuals within their normal places of residence and thus do not prevent household transmission. They may in fact amplify it, by increasing the time household members spend together. If even a small fraction of households have been “seeded” with infection at the time an intervention is implemented, cases may continue to increase for multiple serial intervals. This residual transmission is exacerbated if weak inter-household connections remain, and especially if there are clusters of individuals less able to comply with social distancing measures, for example among communities with a high prevalence of “essential workers”.

Our results show that it is very difficult for interventions which only target transmission outside the house to effectively control the outbreak. Unless these interventions reduce the vast majority of contacts, ongoing transmission in households combined with occasional spillover to other households means that the epidemic may continue to increase long after social distancing begins and when it turns around, declines in cases can be extremely slow. We found that the relative contribution of household and external contacts to transmission was a critical determinant of the overall outcome of social distancing interventions, and the timescale over which effects could be observed. The number of contacts alone was not very informative for predicting intervention efficacy. It is not possible to predict the effect of an intervention that differentially affects household and external contacts by simply estimating the proportional reduction in the total R_0_. For example, even if the component of R_0_ from household transmission alone is greater than 1, infection cannot continue if external connections are substantially weakened. These findings highlight the need for more studies to determine the contribution of different types of contacts to transmission.

The role of household transmission in the spread of COVID-19 is variable across settings. Several studies with detailed contact tracing have attempted to estimate the household “secondary attack rate”, i.e. the probability of transmission per susceptible household member when there is a single infected individual in the house. In a large study in Shenzhen, China, Bi et al estimated this rate at 11% [34]. In Guangzhou, China the estimate was 20% [35], in Beijing 23% [36], in Zhuhai 32% [37], in Seoul, South Korea 16% [38] and in Taiwan, around 5% [39]. In a small German town with a large outbreak due to a superspreading event at a carnival, the household secondary attack rate was closer to 30% but decreased in larger households [40]. Liu et al considered a collection of known clusters involving close contacts in a single gathering (not just household, often group meals), and estimated a 35% secondary attack rate. Lewis et al find a rate of 28% in Wisconsin and Utah [41], while Grijalva et al found 53% in Wisconsin and Tennessee [42]. A recent review by Madewell et al [43] reports values between 4–44%. Curmei et al’s review [44] attempts to collect all these estimates and correct them upwards by accounting for false negative rates of diagnostic tests and for asymptomatic infections, resulting in estimates between ~10–55%. Given that the average household size is relatively small in all these countries (~3 or less), these numbers suggest that infection from outside the house must play a large role in order to explain the overall R_0_ values observed. By varying the relative weight of household vs external contacts, our study examined a range of household secondary attack rates from ~10% to ~65%. Many more studies have examined the role of household transmission in influenza spread, but the results are also equivocal: a review by Tsang et al found that household secondary attack rates varied from 1–40% across studies [45]. A massive cohort study from Japan recently shone some light on this complexity; finding that the risk of household influenza transmission was highly dependent on household structure and on the familial relationship between the primary and secondary case [46].

The networks we use to simulate infection were parameterized based on detailed surveys that used “contact diaries” to track the number of individuals someone interacted with on a randomly chosen day [20,21]. Contacts were generally defined as physical contact or face-to-face conversations, and were meant to capture interactions thought to be important for the spread of droplet-borne respiratory infections like influenza and coronaviruses. Like others, the data we use from these studies is the average number of daily contacts by age of each individual in the pair. However, these surveys also collected information on the duration and frequency of contacts, which could be used in the future to create dynamic networks with a more complex distribution of weights for each types of contact. One limitation of these sorts of surveys is that they are “ego-centric”, meaning that they only inform the distribution of the number of contacts but not the higher order network structure, which can be important for infection spread [26,47]. When we constructed our multi-layer network of external contacts, we used additional information from other studies to include clustering and modularity in our networks. Another limitation is that certain contacts that might be relevant to respiratory infections may be missed in surveys. For example, transmission via contaminated surfaces can occur between individuals who have never directly interacted, as can transmission in group settings where air is shared (e.g. in fitness classes [48] or at restaurants [49]).

There are multiple strategies to augment social distancing policies by reducing household spread, and these have been implemented to different degrees in different countries. We have not considered such combination policies in our analysis, but other models have explored them in detail. Household spread would be reduced by earlier diagnoses of cases (as soon as symptoms begin), proactive testing of exposed household members of cases, options for out-of-home care for individuals with mild symptoms, or better education and assistance with individuals caring for sick household members to avoid infection, for example via household use of face masks and disinfectants [36]. Population-level contact tracing initiatives would obviously also help [50,51]. Early and influential modeling studies that provided the impetus for widespread social distancing policies around the world assumed these policies would be accompanied by case-based interventions that would reduce household spread (e.g. [11,52]), but these measures have not been uniformly adopted, and are still completely absent in most of the United States.

Clearly a major determinant of the efficacy of social distancing policies for COVID-19 is the fractional reduction in contacts, but quantifying this value is difficult. A variety of data sources can provide some information. Surveys conducted in Wuhan and Shanghai, China comparing contacts before and after COVID-19 lockdowns found that the average number of daily contacts was reduced from ~14 in Wuhan and ~20 in Shanghai to ~2, suggesting a more than ~95% reduction in external contacts [53]. In the US, nationally-representative polls in late March/early April found that around three quarters of households were self-isolating [54], and estimated a mean reduction in contacts around 80% [55,56]. Since contact surveys are rare, measures of reductions in human mobility have been used as a proxy for contact rate reductions. Google [57] and Apple [58] provide reports on mobility changes based on user locations sourced from their smartphone mapping apps, as does Cuebiq [59]. Transit, a live-tracking and schedule-aggregating application for public transit, reports changes in service use [60], and SafeGraph publishes changes in foot traffic to different classes of locations [61].

Different measures of mobility often give very different estimates for the efficacy of social distancing interventions. For example, Klein et al found peak US national average reductions in both the radius of mobility and the number of events where device users came within near proximity of each other were about ~50%, whereas communing volume was reduced by ~75% [62,63]. For the same time period, Apple reported ~50% reductions in direction requests, Transit reported ~70% reduction in transit use, Google reported ~40% reductions in visits to retail locations and ~50% in visits to workplaces, and SafeGraph reported an ~80% reduction in foot traffic at bars but only a 20% reduction to grocery stores. Together these results suggest that our simulations assuming ~80% reduction in external contacts—which still often only results in mediocre outcomes—are likely overestimates, if anything, of reality. Wellenius et al attempted to infer the association between these mobility reductions and the particular social distancing policies that caused them. Using Google data they concluded that in the US, initial emergency declarations lead to ~10% reductions, that each additional policy led to another ~20% reduction, and that “shelter-in-place” orders resulted in additional ~30% reductions [64]. By comparing mobility changes to estimates of R_0_ from case counts in countries around the world, Bergman et al estimated that each ~10% reduction in mobility resulted in an ~0.05 reduction in R_0_ [65]. Interestingly, they also found two other results in agreement with our findings here: there was a long delay between reductions in mobility and reductions in inferred R_0_ in many regions, and, the association between reductions in mobility and R_0_ was weaker in regions who implemented large scale contact tracing, which likely reduces household transmission.

Our results highlight the importance of residual contacts between households that remain despite social distancing measures. Many of these contacts are likely to be driven by individuals who must continue to work. Our own analysis of occupations held by residents of Philadelphia, USA, population ~1.5 million, suggested that ~30% of workers had jobs that fell into categories flagged as “essential”. A review by Lan et al of case reports within the first month of the outbreak in multiple countries found that about 15% of these cases were clearly work-related, and that even earlier in the outbreak, this was as high as 50% [66]. A report released by the UK Office of National Statistics found COVID-19 related deaths were much higher in certain occupational groups (e.g. relative risk of 4.5 for male security guards and 2 for female care workers), and the UK Biobank found an 8x higher rate of COVID-19 diagnoses in healthcare workers compared to the general population. Major clusters of infection have occurred in workplaces as varied as call centers [38] and meatpacking plants [67]. The results of our modeling show how risks to essential workers spill over to others, increasing the individual infection risk for workers’ household members and increasing the persistence time of epidemics in the community at large. Given the limited apparent ability to reduce workplace contacts and transmission, reducing household transmission or other external contacts may be even more important. Another strategy we have not considered is selective restructuring of contact networks to increase clustering and decrease mean path length, so that transmission risk is minimized without further reducing contacts [68].

Separate assumptions of our modeling approach could lead our predictions to be slightly pessimistic. We assume a baseline value of R_0_ ~ 3, whereas some other studies have used values between 1.9–2.7 [4–6,51,69]. There are several reasons why we believe those estimates are likely a little too low. Firstly, they tended to assume very short serial intervals and infectious periods, whereas other studies have estimated longer serial intervals [34,70–72], particularly in the absence of quick isolation of mild cases, which is more likely to reflect what is going on in most of the world outside of east Asia. Secondly, those estimates often fit to cases counts that were doubling every 5–6 days, whereas in many settings doubling times were closer to 3 days early in the outbreak [73–76]. Finally, nearly all previous estimates of R_0_ fit a randomly-mixing population (with or without age structure), whereas in our highly structured network population, higher R_0_ values are needed to achieve the same doubling time. R_0_ values as high as 3–6 have been estimated using rigorous model-fitting methods [9,52,72]. With lower R_0_ values, any estimates of the % reduction in external contacts needed to achieve a certain rate of reduction of cases and deaths would be reduced. However, our main qualitative results about delays to epidemic peak and the complex role of household transmission hold.

Our results are not sensitive to our assumptions about the fraction of cases that progress to more serious clinical stages nor to the case fatality risk. However, our estimates for the timing of peak values do depend on the distribution of delays we assume, for example between symptom onset and hospitalization, or between ICU admission and death. There is variability in the estimates of these values across studies (see Supplementary Methods), and these values likely differ by country, depending on the standard of care and the underlying health of the population. While we have considered wide intervals for the interpatient variation in these durations, we have not propagated uncertainty in the distribution of these values. We hope that by providing our code, researchers who are interested in specific contexts where these values may differ significantly can explore those scenarios. There are other factors which influence the delay between implementation of social distancing measures and peak cases and deaths that we have not included in our model. One factor is reporting delays, which may be especially long for deaths in certain regions. Another factor is that there could be a delay between implementation of distancing measures and adoption by a majority of the population.

By including more details of transmission network structure, we are able to examine effects that would not be apparent in well-mixed epidemic models. However, our population structure is still simplistic in many senses. For example, we do not explicitly model the dynamics of certain institutions that have been particularly hard-hit by COVID-19, such as retirement homes and long-term care facilities [77], prisons [78,79], and homeless shelters [80]. Understanding the unique contact networks, transmission risks, host susceptibility, and mortality risks in these populations is an important area for future research. We also do not consider the potential for hospital-acquired transmission and the role of healthcare workers. Doctors, nurses, and other health professionals are reported to make up 5–10% of cases in some regions, and while increased testing is likely one factor driving these rates, it is clear that there are also unique risks to this profession.

Strong social distancing measures tend to be economically costly and in most regions of the world these measures were relaxed to some extent after a few months. In this study we examined a particular partial relaxation strategy in which households form “bubbles” with other households. We predicted that widespread adoption of these bubbles should only occur in the context of decreasing incidence and compensatory reductions in external contacts, in order to maintain epidemic control. Here we imagine that household bubbles are formed voluntarily for social reasons, but households may also be forced to “double-up” when one household experiences eviction from their current residence. The economic recession in the US has led to massive increases in households at risk of eviction, and separate work using a similar model found that evictions could result in substantial increases in cases across cities if the current eviction bans expire [81]. Other modeling studies have explored the impact of generalized relaxation of social distancing on second-wave scenarios [82–85]. Although not the primary focus of this work, when we simulate generalized relaxation we find that cases always begin to increase almost immediately. This is in contrast to the months-long delays between relaxation of interventions and resurgences of cases observed in many parts of Europe and North America in summer through fall 2020. In reality other factors not included in our model are likely to play a role in observed delays post-relaxation, such as a delayed behavioral response to relaxation policies, shifting age distributions of cases, repeated stochastic re-introductions and extinctions, and seasonality. We did observe variable delays until deaths and hospitalizations began to increase again in our simulations, which was explained by the clinical progression times and the degree of relaxation (**S7 Fig**).

Many studies are now attempting to estimate the degree to which different social distancing measures (e.g. school closures, stay-at-home policies) reduce the reproductive ratio or the exponential growth rate of cases. Our results point out a few challenges to these efforts. The long delays we describe in this paper mean that methods that fit simple growth functions to data and look for changes in their values may have trouble identifying effects. If there are a series of interventions that tend to be implemented in similar orders or at similar intervals across settings, and the goal is to estimate the effect of each (e.g. [11,86]), then the delays we describe here could lead to falsely attributing the effect of one intervention to another that occurs later (e.g. see [8] and comments in response). Some of these problems can be avoided by explicit use of mathematical models that take into account the prolonged clinical progression of COVID-19 (e.g. [12,87]), which is the first order cause of these delays. However, our results show that transmission network structure also plays an important role. Importantly, the amount by which overall transmission is reduced by social distancing measures and the delay until effects are seen depends on the relative role of household vs external transmission, which is unknown and may be different by setting.

## Supporting information

S1 Text Supplementary Methods. (PDF)

S1 Fig Creating hierarchical structuring in transmission networks via “neighborhoods”. A) Schematic of the construction of the networks neighborhood clustering. Each household belongs to a mutually-exclusive “neighborhood”, and external connections are preferentially created within the same neighborhood. B-C) Visualizations of connections in the external layer of the two-layer transmission network with and without neighborhood clustering. For ease of visualization, this is plotted for a population size of 1000 with 10 “neighborhoods” (node color) of size 100. In the real simulations, the population size is 10^6^ with 100 neighborhoods of size 10,000. A) The external layer consists of random connections between individuals, irrespective of their neighborhoods. B) 90% of external connections are preferentially attached to individuals within their own neighborhood, creating more intermediate-scale clustering in the network.
(TIF)

S2 Fig Dynamics pre and post social distancing interventions in network-structured populations with household and external transmission, with neighborhood clustering. A) Multi-layer network of transmission. Individuals have contacts within their households and with others outside the household, which preferentially occur within a local neighborhood (S1 Fig). Household and external contacts may have different weights (e.g. different likelihood of transmission), due to for example different levels of physical contact or time spent together per day. Social distancing interventions (red X) remove or decrease the weight of external contacts. B) Distribution of household sizes. C) Distribution of the # of contacts (degree) within the household and outside the household. D) The contribution of household and external spread to the total R_0_ value as a function of the relative weight of external contacts. E)-F) Simulated time course of different clinical stages of infection under an intervention with efficacy of 100% (E) or 80% (F) at reducing external contacts, when household and external contacts have equal weight. Black dotted line shows the time the intervention began. G) The role of the relative importance of household vs external contacts in determining the outcome of the intervention, measured by the size of the epidemic. Epidemic final size is defined as the percent of the population who have recovered by day 300. H-J) Same as above but under the scenario where the weight of household contacts doubles post-intervention (*w*_*HH*_ → 2*w*_*HH*_, due to increased time spent in house). K) The household secondary attack rate, defined as the probability of transmission per susceptible household member when there is a single infected individual in the house, as a function of the relative weight of external contacts. L) The percent of households which are “seeded” with infection at the time the intervention was implemented (i.e. have at least one infected individual). In all scenarios the overall infection prevalence at the time intervention was started was identical.
(TIF)

S3 Fig Clustered vs uniform adoption of social distancing measures, with lower efficacy. A) Schematic of the multi-layer network created to more realistically capture non-household contacts and how they are altered by social distancing measures. In each layer, the degree distribution and level of clustering were chosen to match data. The “community” layer represents any other contact not fitting in the other four categories. Colors of nodes represent four broad age groups that determine network membership and structure: preschool-aged (pink), school-aged (purple), working-aged (blue) and elderly (green). B)—C) Simulated time courses of infection in the presence of social distancing intervention with random (B) vs clustered (C) adherence to measures. In both cases, all school connections were deleted post-intervention and 70% of connections were uniformly deleted at random in the social and community layers. In B) 70% of work connections were uniformly deleted whereas, in C) 70% of workplaces were dissolved, leading to clusters of disconnected vs connected individuals in the work layer of the network. The effective intervention efficacy for all layers combined was ~ 75% in both scenarios. Black dotted line shows the time the intervention began.
(TIF)

S4 Fig Clustered vs uniform adoption of social distancing measures, with neighborhood clustering. A) Schematic of the multi-layer network created to more realistically capture non-household contacts and how they are altered by social distancing measures. In each layer, the degree distribution and level of clustering were chosen to match data. The “community” layer represents any other contact not fitting in the other four categories. Colors of nodes represent four broad age groups that determine network membership and structure: preschool-aged (pink), school-aged (purple), working-aged (blue) and elderly (green). Community and school connections occurred within local neighborhoods. B)—E) Simulated time courses of infection in the presence of social distancing intervention with random (B,D) vs clustered (C,E) adherence to measures. In both cases, all school connections were deleted post-intervention and 85% (top) or 70% (bottom) of connections were uniformly deleted at random in the social and community layers. In B) 85% (top) or 70% (bottom) of work connections were uniformly deleted whereas, in C) 85% (top) or 70% (bottom) of workplaces were dissolved, leading to clusters of disconnected vs connected individuals in the work layer of the network. The effective intervention efficacy for all layers combined was ~ 88% (top) or ~75% (bottom) in both scenarios. Black dotted line shows the time the intervention began.
(TIF)

S5 Fig Effect of partially relaxing intervention by forming household bubbles, with neighborhood clustering. Some time after a social distancing intervention was implemented, each household merges with another random household irrespective of their “neighborhood”. In each resulting two-household “bubble”, all individuals are connected to all other individuals. A)-D) Simulated time courses of infection before and after social distancing interventions (with 80% vs 90% intervention efficacy) and after partial-relaxation by household merging. Top row: External contacts of individuals were unchanged after two households were merged, such that overall number of contacts increased. Bottom row: External contacts for individuals were reduced after two households were merged, such that overall number of contacts remained unchanged. In all cases, intervention was started 43 days after the onset of the epidemic (first black dotted line) and was relaxed after two months (60 days, second black dotted line).
(TIF)

S6 Fig Effect of partially relaxing intervention by forming household bubbles, with neighborhood clustering. Some time after a social distancing intervention was implemented, each household merges with another household from their own “neighborhood”. In each resulting two-household “bubble”, all individuals are connected to all other individuals. A)-D) Simulated time courses of infection before and after social distancing interventions (with 80% vs 90% intervention efficacy) and after partial-relaxation by household merging. Top row: External contacts of individuals were unchanged after two households were merged, such that overall number of contacts increased. Bottom row: External contacts for individuals were reduced after two households were merged, such that overall number of contacts remained unchanged. In all cases, intervention was started 43 days after the onset of the epidemic (first black dotted line) and was relaxed after two months (60 days, second black dotted line).
(TIF)

S7 Fig Effects of generalized relaxation of social distancing measures. Simulated time course of different clinical stages of infection under an initial intervention that reduces contacts (first black dashed line) and subsequent relaxation of that intervention (second black dashed line). Plots show daily incidence as a fraction of the total population. Solid line is mean and shaded areas are 5th and 95th percentile. Results are shown for different values of the efficacy of the initial intervention, efficacy during relaxation, and the timing of relaxation. The efficacy of the initial intervention and relaxation are defined as the % reduction in external contacts. In all plots, household and external contacts have equal weight. Plot annotations report the median time post-relaxation until different metrics of disease burden begin to increase—the daily incidence of new cases, current hospitalizations, or daily deaths. If deaths go to zero under an intervention, we instead report median time post-intervention to first death.
(TIF)

S8 Fig Time-varying effective reproduction number under social distancing. Top row: Simulated time course of different clinical stages of infection under an intervention (first black dashed line) that reduces external contacts by 80%, for different values of the relative weight of external contacts. Plots show daily incidence (for a total population of 1 million). Solid line is mean and shaded areas are 5th and 95th percentile of 100 simulations. Second row: Effective reproduction number (*R*_*t*_) over time calculated using the daily incidence of new infections (I_1_) for each simulation, calculated using the EpiEstim method with a 7-day window. The *R*_*t*_ value plotted for day *t* is calculated using timepoints before *t* only. Third row: Same but using the daily incidence of new hospitalizations (I_*2*_). Bottom row: The first timepoint after the intervention that *R*_*t*_ <1. Dots show the median and bars represent 5th and 95th percentiles.
(TIF)
