# Supplementary figures and images for: Dynamics of COVID-19 under social distancing measures are driven by transmission network structure
Source: PLoS Comput Biol. 2021 Feb 3;17(2):e1008684. doi: 10.1371/journal.pcbi.1008684 (PMC7886148; doi:10.1371/journal.pcbi.1008684)

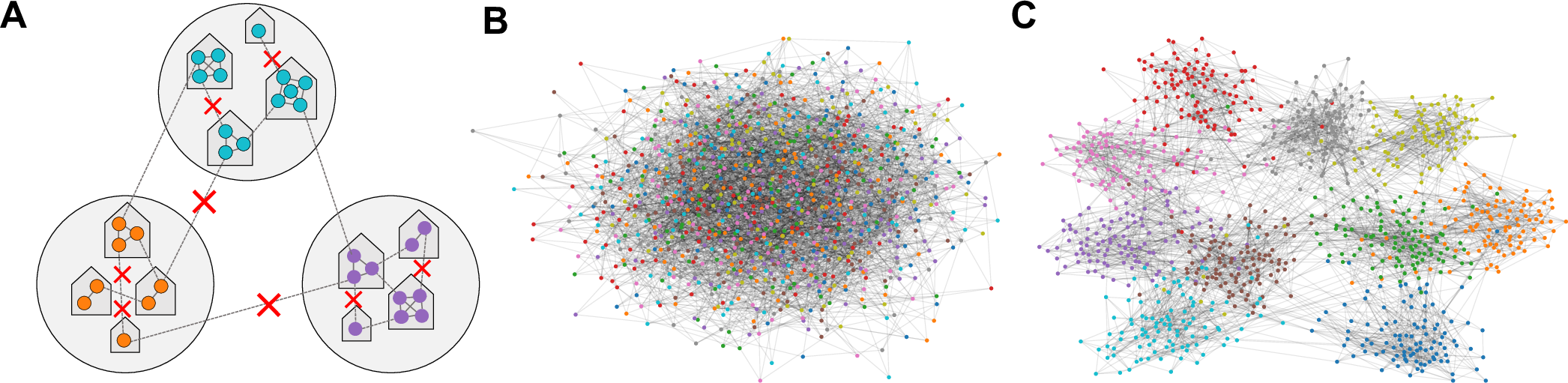

Supplement: S1 Fig — A) Schematic of the construction of the networks neighborhood clustering. Each household belongs to a mutually-exclusive “neighborhood”, and external connections are preferentially created within the same neighborhood. B-C) Visualizations of connections in the external layer of the two-layer transmission network with and without neighborhood clustering. For ease of visualization, this is plotted for a population size of 1000 with 10 “neighborhoods” (node color) of size 100. In the real simulations, the population size is 106 with 100 neighborhoods of size 10,000. A) The external layer consists of random connections between individuals, irrespective of their neighborhoods. B) 90% of external connections are preferentially attached to individuals within their own neighborhood, creating more intermediate-scale clustering in the network. (TIF) [file pcbi.1008684.s002.tif]

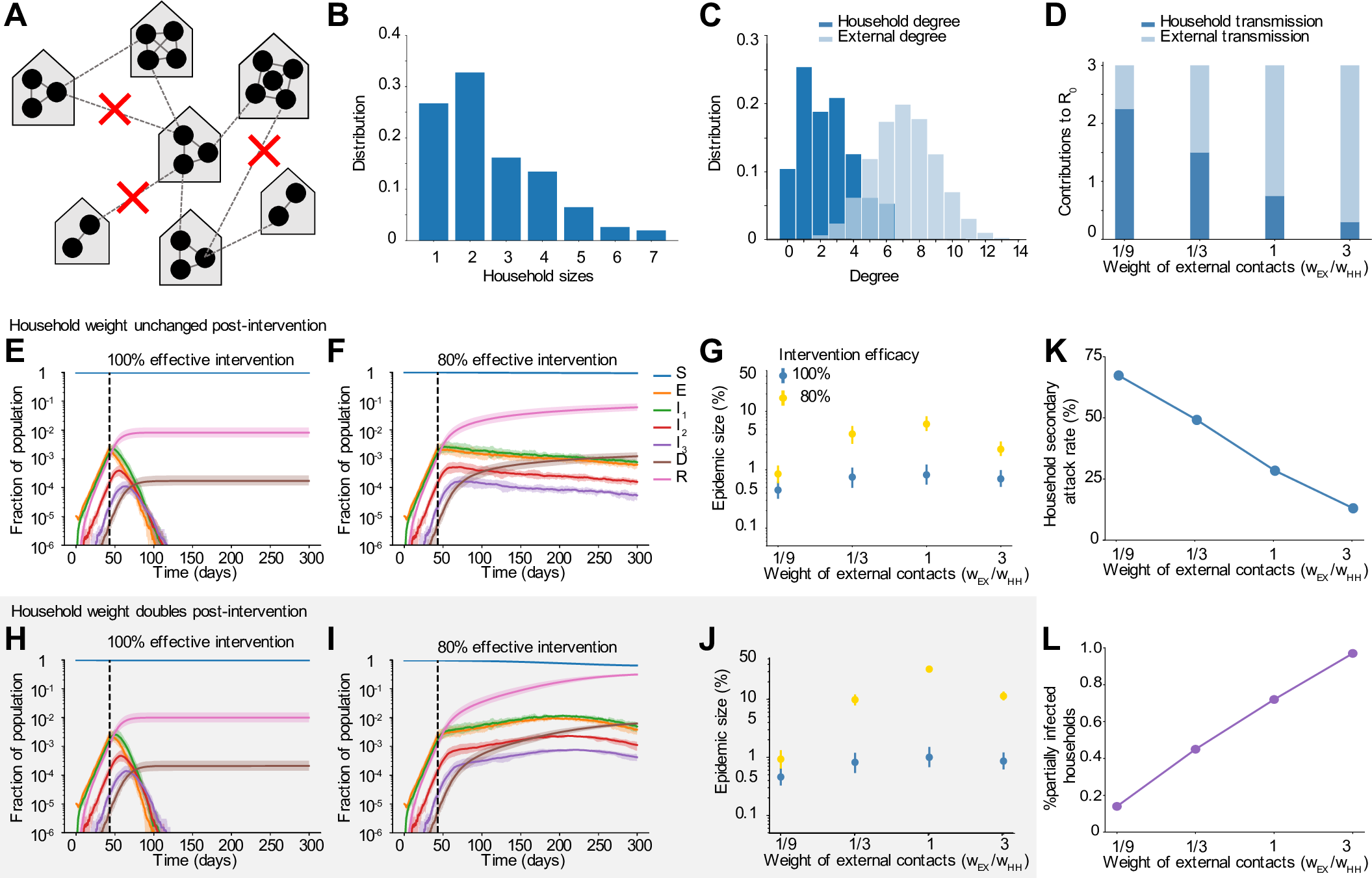

Supplement: S2 Fig — A) Multi-layer network of transmission. Individuals have contacts within their households and with others outside the household, which preferentially occur within a local neighborhood (S1 Fig). Household and external contacts may have different weights (e.g. different likelihood of transmission), due to for example different levels of physical contact or time spent together per day. Social distancing interventions (red X) remove or decrease the weight of external contacts. B) Distribution of household sizes. C) Distribution of the # of contacts (degree) within the household and outside the household. D) The contribution of household and external spread to the total R0 value as a function of the relative weight of external contacts. E)-F) Simulated time course of different clinical stages of infection under an intervention with efficacy of 100% (E) or 80% (F) at reducing external contacts, when household and external contacts have equal weight. Black dotted line shows the time the intervention began. G) The role of the relative importance of household vs external contacts in determining the outcome of the intervention, measured by the size of the epidemic. Epidemic final size is defined as the percent of the population who have recovered by day 300. H-J) Same as above but under the scenario where the weight of household contacts doubles post-intervention (wHH → 2wHH, due to increased time spent in house). K) The household secondary attack rate, defined as the probability of transmission per susceptible household member when there is a single infected individual in the house, as a function of the relative weight of external contacts. L) The percent of households which are “seeded” with infection at the time the intervention was implemented (i.e. have at least one infected individual). In all scenarios the overall infection prevalence at the time intervention was started was identical. (TIF) [file pcbi.1008684.s003.tif]

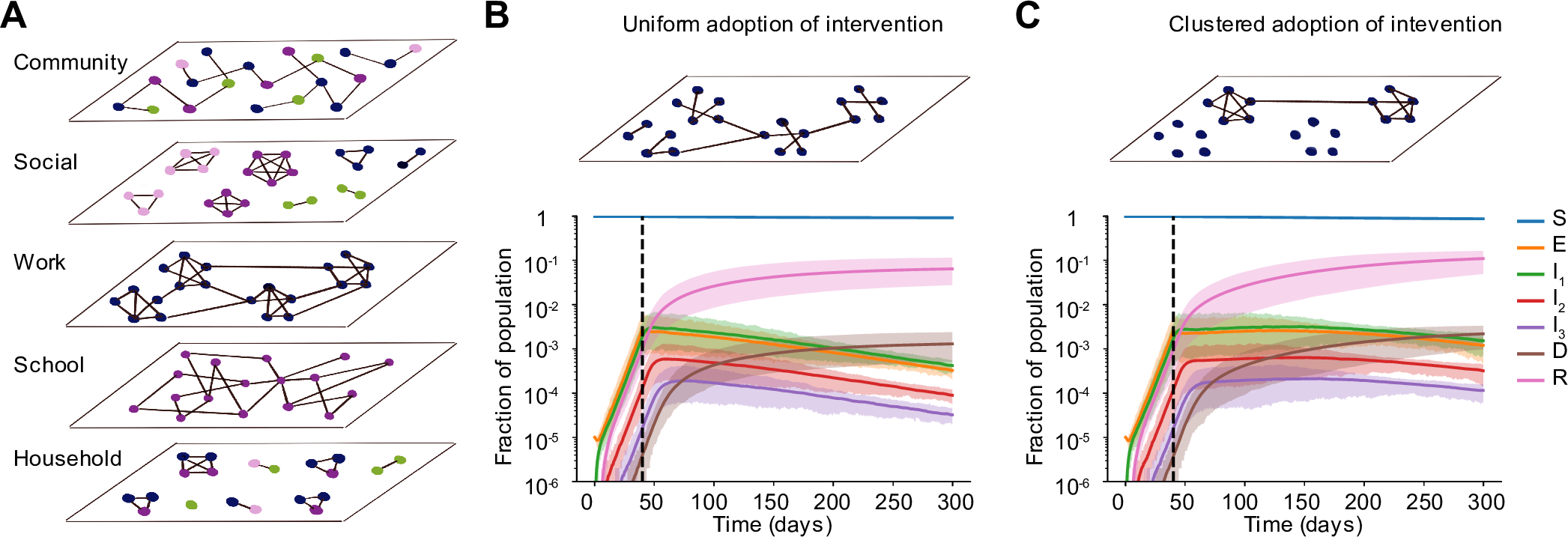

Supplement: S3 Fig — A) Schematic of the multi-layer network created to more realistically capture non-household contacts and how they are altered by social distancing measures. In each layer, the degree distribution and level of clustering were chosen to match data. The “community” layer represents any other contact not fitting in the other four categories. Colors of nodes represent four broad age groups that determine network membership and structure: preschool-aged (pink), school-aged (purple), working-aged (blue) and elderly (green). B)—C) Simulated time courses of infection in the presence of social distancing intervention with random (B) vs clustered (C) adherence to measures. In both cases, all school connections were deleted post-intervention and 70% of connections were uniformly deleted at random in the social and community layers. In B) 70% of work connections were uniformly deleted whereas, in C) 70% of workplaces were dissolved, leading to clusters of disconnected vs connected individuals in the work layer of the network. The effective intervention efficacy for all layers combined was ~ 75% in both scenarios. Black dotted line shows the time the intervention began. (TIF) [file pcbi.1008684.s004.tif]

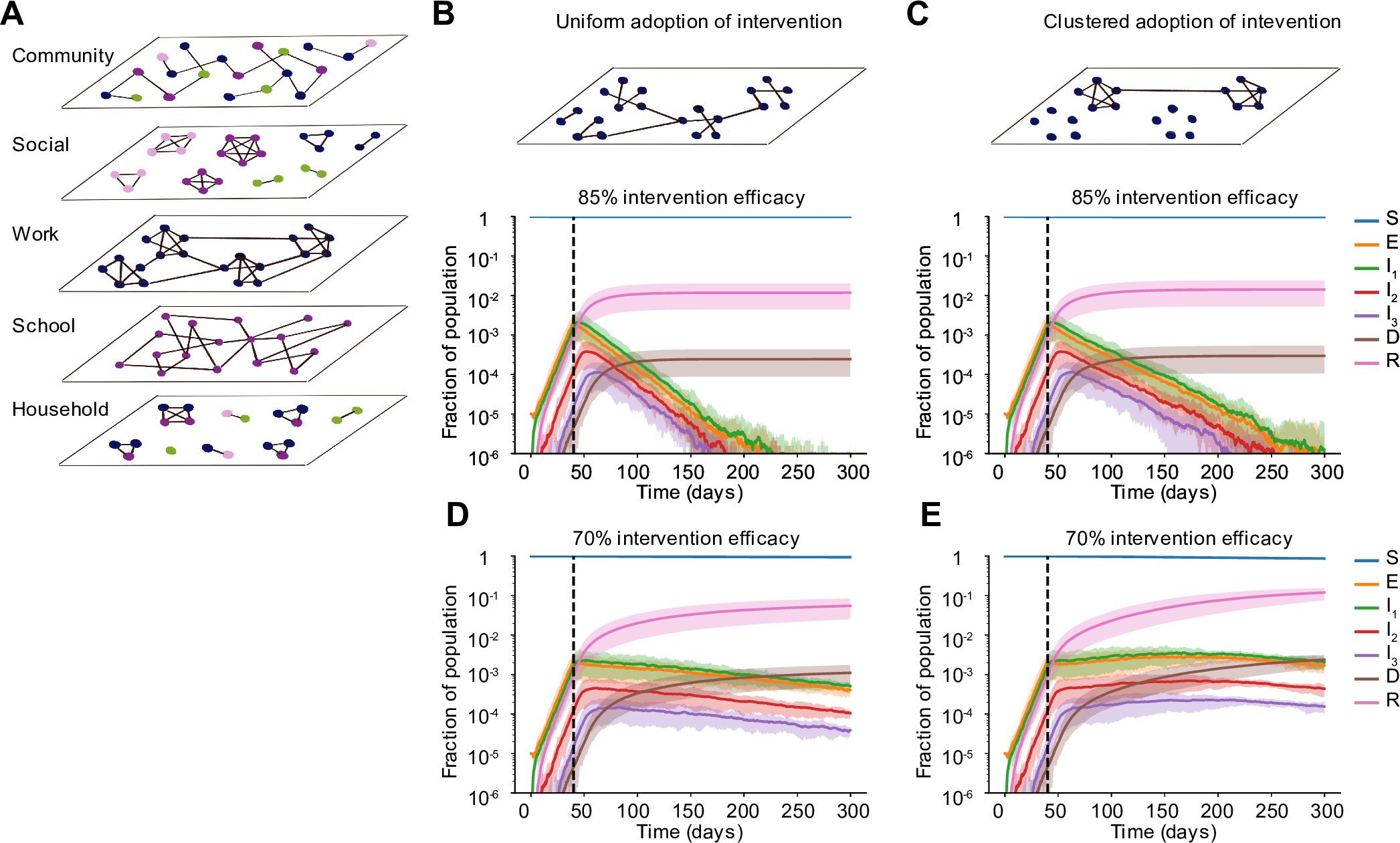

Supplement: S4 Fig — A) Schematic of the multi-layer network created to more realistically capture non-household contacts and how they are altered by social distancing measures. In each layer, the degree distribution and level of clustering were chosen to match data. The “community” layer represents any other contact not fitting in the other four categories. Colors of nodes represent four broad age groups that determine network membership and structure: preschool-aged (pink), school-aged (purple), working-aged (blue) and elderly (green). Community and school connections occurred within local neighborhoods. B)—E) Simulated time courses of infection in the presence of social distancing intervention with random (B,D) vs clustered (C,E) adherence to measures. In both cases, all school connections were deleted post-intervention and 85% (top) or 70% (bottom) of connections were uniformly deleted at random in the social and community layers. In B) 85% (top) or 70% (bottom) of work connections were uniformly deleted whereas, in C) 85% (top) or 70% (bottom) of workplaces were dissolved, leading to clusters of disconnected vs connected individuals in the work layer of the network. The effective intervention efficacy for all layers combined was ~ 88% (top) or ~75% (bottom) in both scenarios. Black dotted line shows the time the intervention began. (TIF) [file pcbi.1008684.s005.tif]

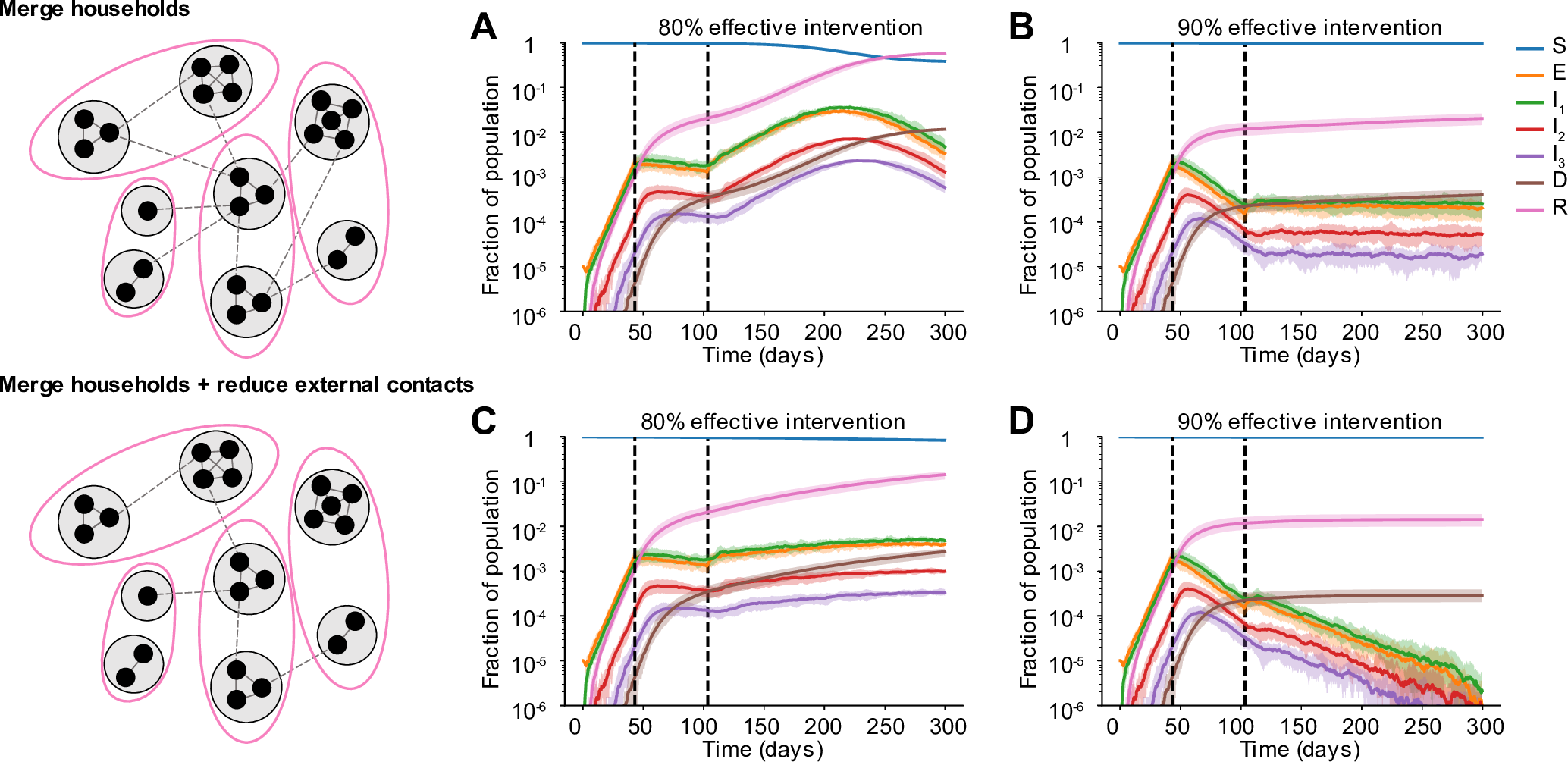

Supplement: S5 Fig — Some time after a social distancing intervention was implemented, each household merges with another random household irrespective of their “neighborhood”. In each resulting two-household “bubble”, all individuals are connected to all other individuals. A)-D) Simulated time courses of infection before and after social distancing interventions (with 80% vs 90% intervention efficacy) and after partial-relaxation by household merging. Top row: External contacts of individuals were unchanged after two households were merged, such that overall number of contacts increased. Bottom row: External contacts for individuals were reduced after two households were merged, such that overall number of contacts remained unchanged. In all cases, intervention was started 43 days after the onset of the epidemic (first black dotted line) and was relaxed after two months (60 days, second black dotted line). (TIF) [file pcbi.1008684.s006.tif]

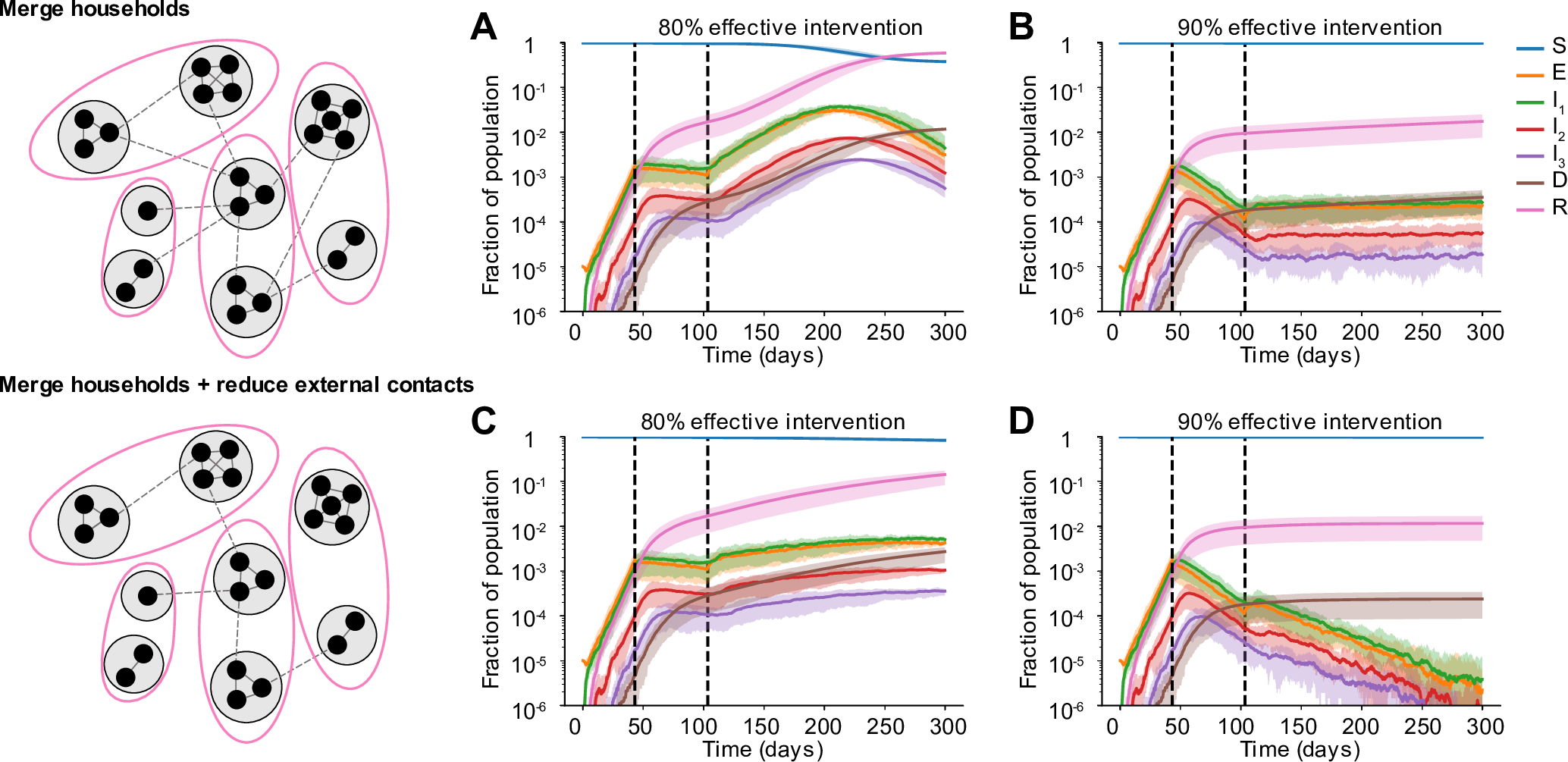

Supplement: S6 Fig — Some time after a social distancing intervention was implemented, each household merges with another household from their own “neighborhood”. In each resulting two-household “bubble”, all individuals are connected to all other individuals. A)-D) Simulated time courses of infection before and after social distancing interventions (with 80% vs 90% intervention efficacy) and after partial-relaxation by household merging. Top row: External contacts of individuals were unchanged after two households were merged, such that overall number of contacts increased. Bottom row: External contacts for individuals were reduced after two households were merged, such that overall number of contacts remained unchanged. In all cases, intervention was started 43 days after the onset of the epidemic (first black dotted line) and was relaxed after two months (60 days, second black dotted line). (TIF) [file pcbi.1008684.s007.tif]

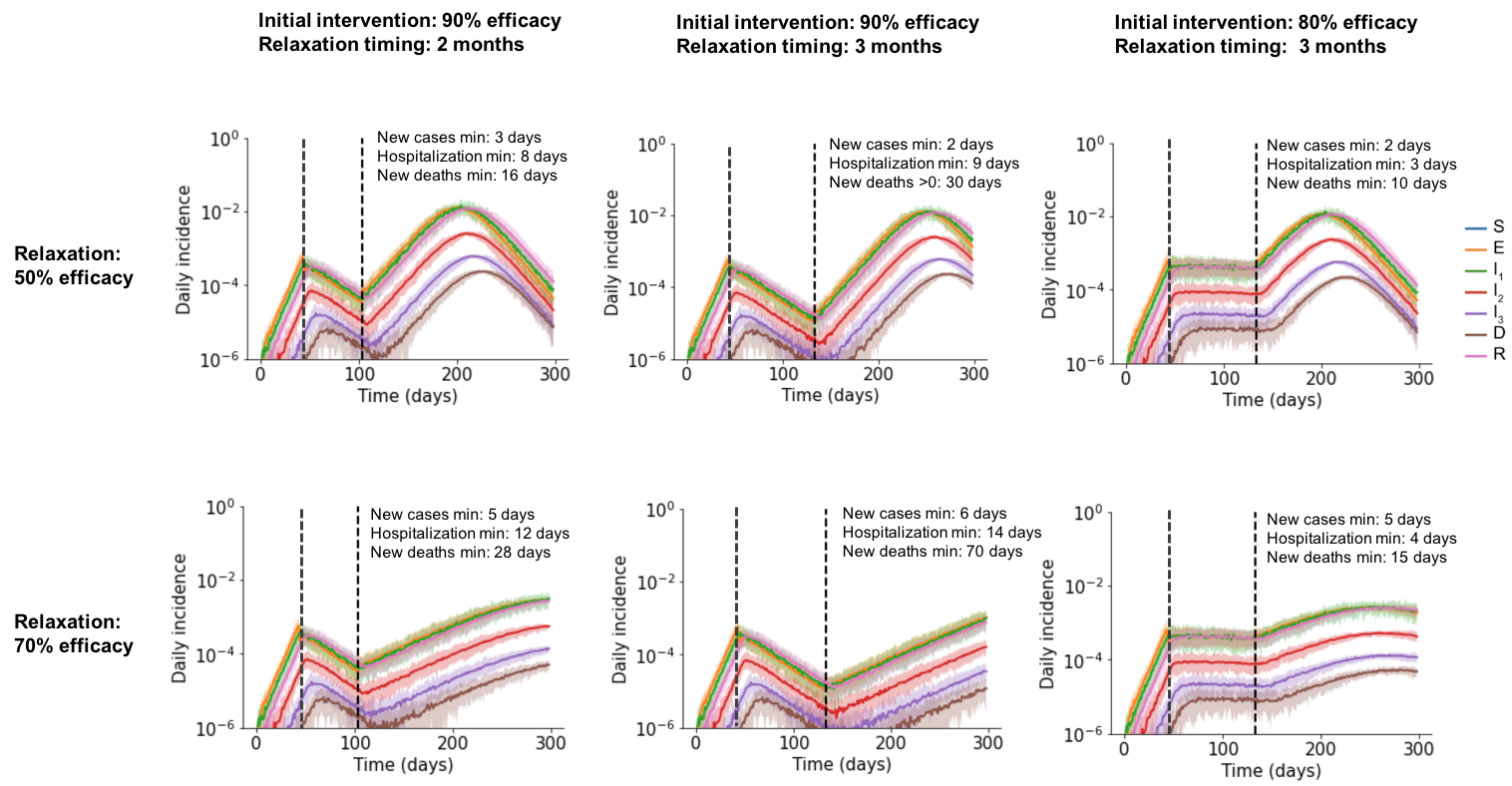

Supplement: S7 Fig — Simulated time course of different clinical stages of infection under an initial intervention that reduces contacts (first black dashed line) and subsequent relaxation of that intervention (second black dashed line). Plots show daily incidence as a fraction of the total population. Solid line is mean and shaded areas are 5th and 95th percentile. Results are shown for different values of the efficacy of the initial intervention, efficacy during relaxation, and the timing of relaxation. The efficacy of the initial intervention and relaxation are defined as the % reduction in external contacts. In all plots, household and external contacts have equal weight. Plot annotations report the median time post-relaxation until different metrics of disease burden begin to increase—the daily incidence of new cases, current hospitalizations, or daily deaths. If deaths go to zero under an intervention, we instead report median time post-intervention to first death. (TIF) [file pcbi.1008684.s008.tif]

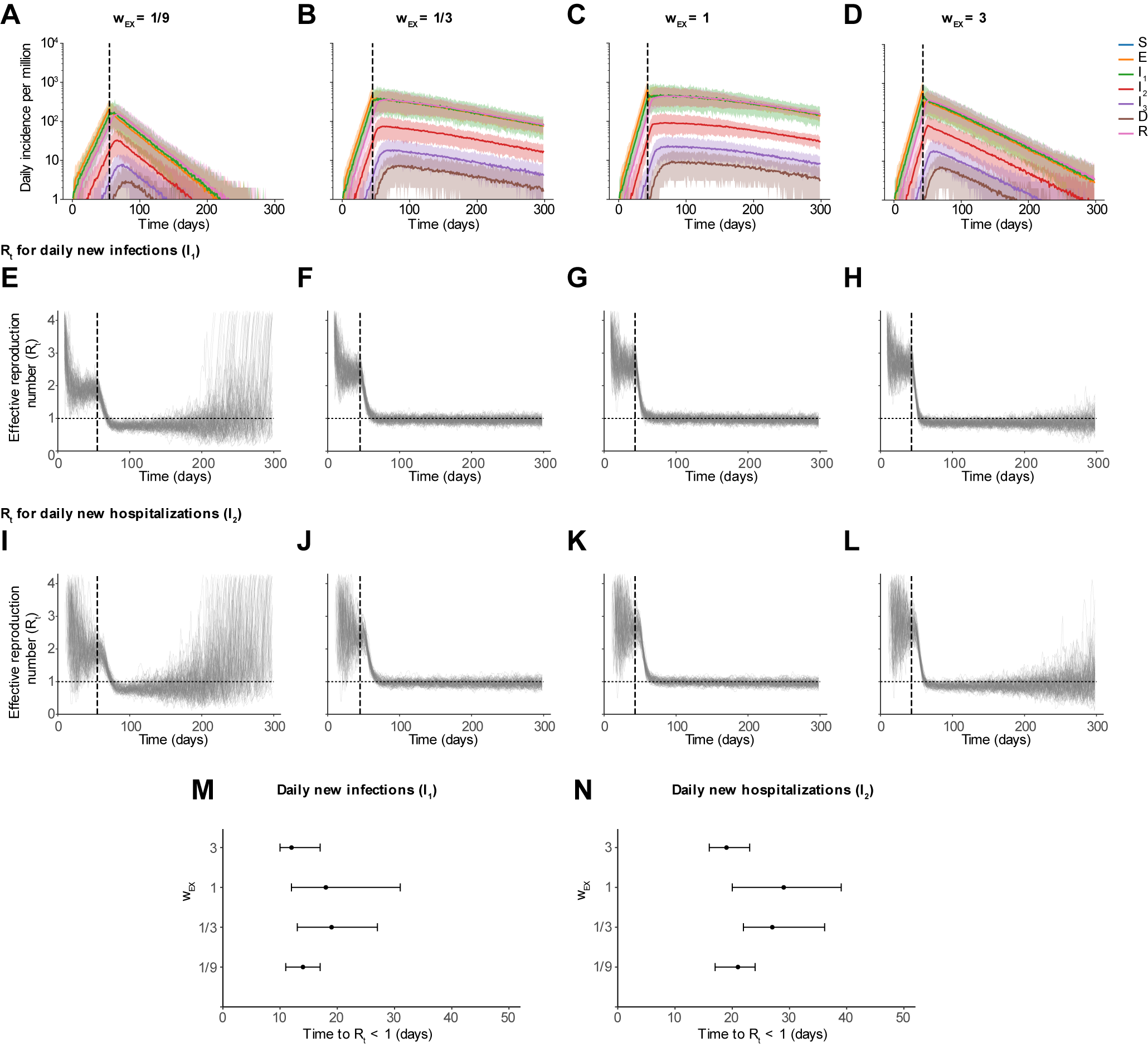

Supplement: S8 Fig — Top row: Simulated time course of different clinical stages of infection under an intervention (first black dashed line) that reduces external contacts by 80%, for different values of the relative weight of external contacts. Plots show daily incidence (for a total population of 1 million). Solid line is mean and shaded areas are 5th and 95th percentile of 100 simulations. Second row: Effective reproduction number (Rt) over time calculated using the daily incidence of new infections (I1) for each simulation, calculated using the EpiEstim method with a 7-day window. The Rt value plotted for day t is calculated using timepoints before t only. Third row: Same but using the daily incidence of new hospitalizations (I2). Bottom row: The first timepoint after the intervention that Rt <1. Dots show the median and bars represent 5th and 95th percentiles. (TIF) [file pcbi.1008684.s009.tif]
